# Identification of Specific Biomarkers and Pathways in the Treatment Response of Infliximab for Inflammatory Bowel Disease: In-Silico Analysis

**DOI:** 10.3390/life13030680

**Published:** 2023-03-02

**Authors:** Rachid Kaddoura, Hardik Ghelani, Fatma Alqutami, Hala Altaher, Mahmood Hachim, Reem Kais Jan

**Affiliations:** Department of Basic Science, College of Medicine, Mohammed Bin Rashid University of Medicine and Health Sciences, Dubai P.O. Box 505055, United Arab Emirates

**Keywords:** inflammatory bowel disease, in-silico, transcriptomics, differentially expressed gene analysis, infliximab treatment response

## Abstract

Background: Inflammatory bowel disease (IBD) is characterized by chronic inflammation of the gastrointestinal tract. In biological therapy, infliximab became the first anti-tumor necrosis factor (TNF) agent approved for IBD. Despite this success, infliximab is expensive, often ineffective, and associated with adverse events. Prediction of infliximab resistance would improve overall potential outcomes. Therefore, there is a pressing need to widen the scope of investigating the role of genetics in IBD to their association with therapy response. Methods: In the current study, an in-silico analysis of publicly available IBD patient transcriptomics datasets from Gene Expression Omnibus (GEO) are used to identify subsets of differentially expressed genes (DEGs) involved in the pathogenesis of IBD and may serve as potential biomarkers for Infliximab response. Five datasets were found that met the inclusion criteria. The DEGs for datasets were identified using limma R packages through the GEOR2 tool. The probes’ annotated genes in each dataset intersected with DGEs from all other datasets. Enriched gene Ontology Clustering for the identified genes was performed using Metascape to explore the possible connections or interactions between the genes. Results: 174 DEGs between IBD and healthy controls were found from analyzing two datasets (GSE14580 and GSE73661), indicating a possible role in the pathogenesis of IBD. Of the 174 DEGs, five genes (SELE, TREM1, AQP9, FPR2, and HCAR3) were shared between all five datasets. Moreover, these five genes were identified as downregulated in the infliximab responder group compared to the non-responder group. Conclusions: We hypothesize that alteration in the expression of these genes leads to an impaired response to infliximab in IBD patients. Thus, these genes can serve as potential biomarkers for the early detection of compromised infliximab response in IBD patients.

## 1. Introduction

Inflammatory bowel disease (IBD), encompassing ulcerative colitis (UC) and Crohn’s disease (CD), is a relapsing-remitting inflammatory condition of the gastrointestinal tract. It mainly affects the intestines and is characterized by mucosal and submucosal chronic inflammation, loss of epithelial tight junction integrity, and dysregulated immune response [1]. IBD affects both genders equally, and its incidence has continuously risen in the last few decades [2]. Patients with IBD typically present with a broad spectrum of symptoms, including abdominal pain, alternating bouts of diarrhea and constipation, and hematochezia [1]. The etiology of IBD is not entirely understood; however, it is known to be multifactorial, involving the interaction of three factors that appear to play a significant role: genetics, immune response, and environmental triggers [3]. Many studies have focused on the role of genetics in the development and progression of IBD [4,5,6]. Monozygotic twins have been shown to have up to 58% concordance to develop IBD; in addition, there is a four-to-six-fold increased risk of developing the condition in patients with first-degree relatives having the disease [4,7]. The genetic background and some of the pathogenetic pathways have been explored by genome-wide association studies (GWAS), which has paved the way for pathway-specific therapy. However, those studies identified a very high number of potential gene variants with a low-risk score for each [8,9]. Hence, there is evidence and a pressing need to investigate the specific genes involved and their interactions, as well as the protein expression across the IBD spectrum, to further our understanding of the etiology and pathophysiology of IBD. The treatment options of a patient diagnosed with IBD are limited, with no medical or surgical cure yet developed [10]. The conventional approach deals with managing symptoms that arise from the disease through pharmacotherapy, which includes anti-inflammatories, corticosteroids for acute flares [11], immunomodulators like methotrexate, and antibiotics [12]. However, conventional medications are poorly specific and can compromise immunity, predisposing patients to infections and a higher chance of neoplastic conditions [13]. Furthermore, many patients experience primary non-response or secondary loss of response to therapy, cycling through several treatments to achieve remission, an error-prone process [14]. 

Two decades ago, introducing the first anti-tumor necrosis factor (anti-TNF) drug, infliximab, revolutionized the medical treatment for moderate to severe IBD, showing high efficacy and a good safety profile [15]. Infliximab is a monoclonal antibody that neutralizes TNF and prevents it from binding to its receptor, thereby reducing the release of pro-inflammatory cytokines that cause tissue damage and inflammation [16]. Various studies, including randomized controlled studies, have shown the efficacy of infliximab in treating IBD with favorable long-term therapeutic outcomes [17,18]. Nonetheless, 30% of patients show no response upon induction, and up to 50% become unresponsive with time [15].

The variation of treatment response can be due to the dynamics of the pathophysiology of IBD; therefore, it is essential to look at differential gene expression in IBD patients, aiming for better prediction of therapy outcomes and more personalized treatment. Transcriptomics data of preclinical and clinical samples are frequently used in biomedical research to identify biomarkers and new therapeutic targets and understand disease pathogenesis. For IBD research, analysis is best performed on mucosal biopsies obtained during sigmoidoscopy or colonoscopy, commonly performed for histopathologic evaluation of disease activity and molecular analysis [19,20].

This current study presents an in silico reanalysis of publicly available GEO transcriptomics microarray datasets obtained from IBD patients (treated with infliximab) and their healthy counterparts. This reanalysis aimed to identify subsets of differentially expressed genes (DEGs) that could be significant players in the IBD pathogenesis and can affect the therapeutic response to infliximab. By grouping IBD patients’ transcriptomics profiles and comparing them to healthy controls, genes considered to be key players in the pathogenesis and progression of IBD could be identified. Subsequently, grouping transcriptomic data of patients undertaking infliximab therapy according to their response, DEGs involved in treatment response were identified and further explored by enriched ontology clustering using Metascape to find possible interactions between the identified genes. Detecting these genes can shed light on relevant disease mechanisms of IBD and aid in developing targeted treatment regimes.

## 2. Materials and Methods

### 2.1. Transcriptomic Data Selection

**Figure 1:**
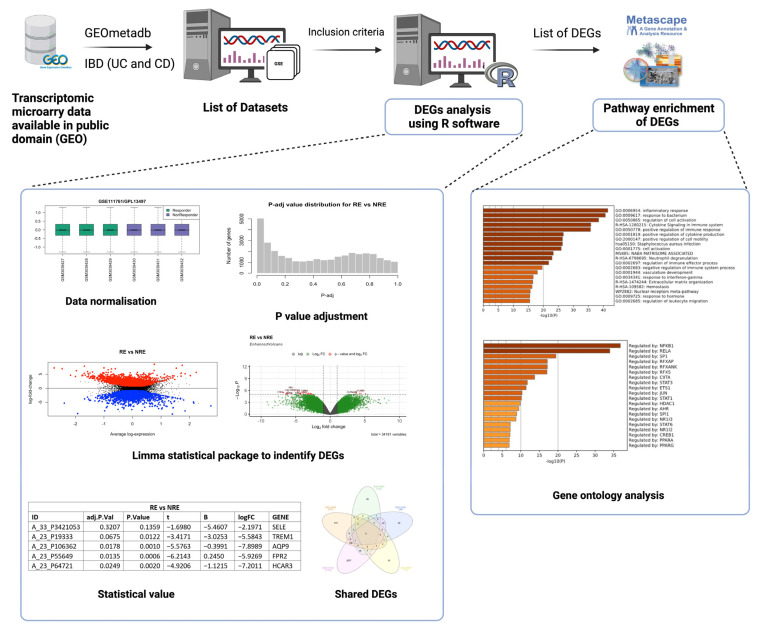
Workflow chart for selection of transcriptomic datasets, differentially expressed genes (DEGs) analysis using R software, and gene ontology analysis (created on BioRender).

A comprehensive search on National Centre for Biotechnology Gene Expression Omnibus (GEO) (https://www.ncbi.nlm.nih.gov/geo/, accessed on 15 November 2022) was carried out using the keywords inflammatory bowel disease, ulcerative colitis, and Crohn’s disease (Figure 1). Each keyword was searched individually, and for consistency purposes, datasets with the following criteria were included to filter down the results to include only datasets that meet the following criteria: (1) Acquired from homo sapiens; (2) Of the study type “expression profiling by microarray”; (3) Treatment included “infliximab”; (4) Containing clinical information regarding the treatment response to infliximab; (5) Containing three samples or more. Only five of the datasets available on IBD fulfilled the inclusion criteria. Table 1 lists the datasets that fulfilled the inclusion criteria.

### 2.2. Identification of Common DEGs and Pathway Enrichment Analysis

**Figure 2:**
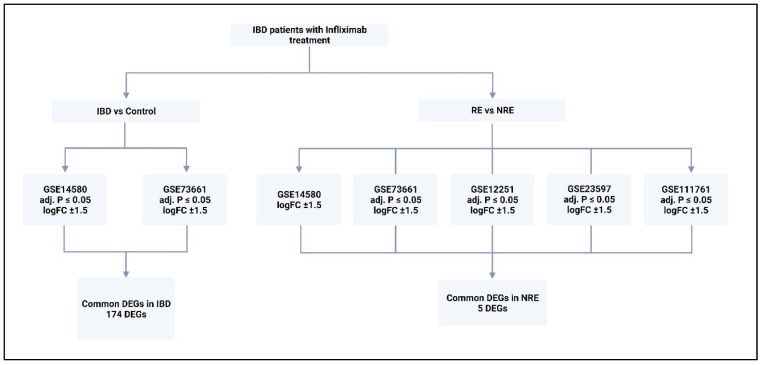
The type of analysis carried out on each dataset and the criteria for DEGs selection. Five datasets were analyzed; two were analyzed by comparing control with IBD, and comparing responders to infliximab with non-responders. All generated DEGs were analyzed using Metascape online tool. RE = responders, NRE = non-responders, logFC = log fold change. DEGs = differentially expressed genes.

The analysis to retrieve the DEGs was performed using the GEO query and limma R packages through the GEO2R tool and R Studio software (R version 4.2.2) for each dataset as described previously [21]. GSE14580 and GSE73661 were the only two datasets with a healthy control group. Therefore, their samples were grouped into IBD patients and healthy controls to identify DEGs based on the disease state. All five datasets were analyzed by comparing infliximab responders (RE) to infliximab non-responders (NRE). The GEO2R input code was used as the basis of this analysis but was further adjusted and modified accordingly in R studio for reanalysis. Prior to reanalysis, all datasets were log-transformed and normalized. The ‘limma’ package was used for the statistical analysis of DEGs and for applying precision weights. Statistical analysis was carried out using the ‘ebayes’ function from the ‘limma’ package, and all outputs were exported. DEG output files must contain a Probe ID, adjusted *p*-value, *p*-value, moderated t-statistic, B-statistic, log fold change (logFC), gene symbol, and gene name (Figure 1). Genes in each dataset analysis were considered significant when adjusting *p*-value ≤ 0.05 and if the logFC was at least ±1.5. If a dataset fails to comply with one of these criteria or does not reach a minimum threshold of 100 DEGs, adjustments were made to include at least one criterion (Figure 2; Supplementary Table S1). Gene Ontology and pathway enrichment analysis were carried out using Metascape (http://metascape.org, accessed on 23 November 2022) to identify the pathways and transcription factors of these DEGs for each dataset analysis. Furthermore, IBD vs.. control DEGs (from GSE14580 and GSE73661) were intersected to identify common DEGs between the two datasets. The RE vs. NRE DEGs were intersected to identify DEGs common in non-responders between all five datasets. 

### 2.3. Further Analysis of Common DEGs

For each dataset, the normalized expression values of the common DEGs were re-analyzed and plotted in GraphPad Prism software. A heatmap was generated using log fold change values of the DEGs in each dataset. Dot plots for the normalized log-transformed expression of each of these DEGs in the selected conditions were carried out, and an unpaired student *t*-test was carried out on each gene within each dataset. Genes with *p* < 0.05 were considered statistically significant.

## 3. Results

### 3.1. DEGs in IBD vs. Control

**Figure 3:**
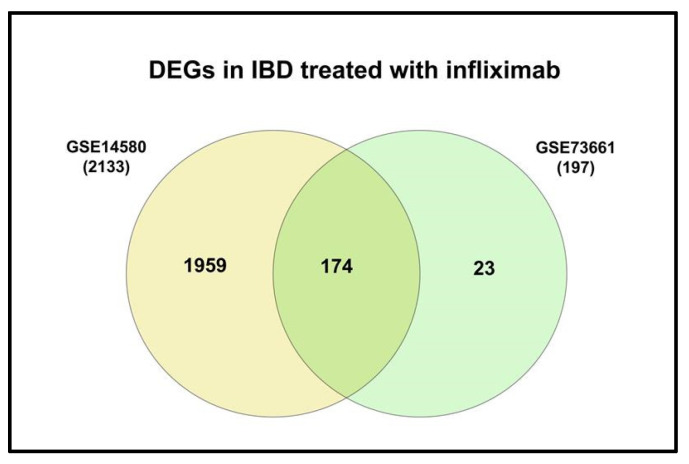
Venn diagram of the shared DEGs in IBD from GSE14480 and GSE73661.

Only two of the five selected datasets (GSE14580 and GSE73661) included a healthy control group. Therefore, they were used to investigate DEGs between IBD and healthy groups; GSE14580 revealed 2133 DEGs, while GSE73661 revealed 197 DEGs. Subsequently, DEGs from both datasets were intersected to generate a short list of 174 genes identified as key players in IBD (Figure 3; Supplementary Tables S2 and S3; Supplementary Figure S1). 

### 3.2. DEGs in Infliximab Responder vs. Non-Responder 

**Figure 4:**
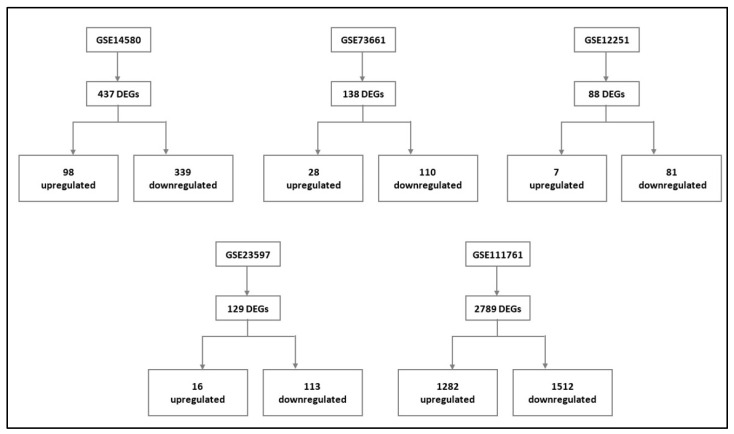
Differentially expressed genes (DEGs) of each dataset and their respective expression (upregulated and downregulated) in the infliximab responder group compared to the infliximab non-responder group (For details, please see Supplementary File of data analysis of the individual dataset, Tables S4–S8, Figures S2–S6).

**Figure 5:**
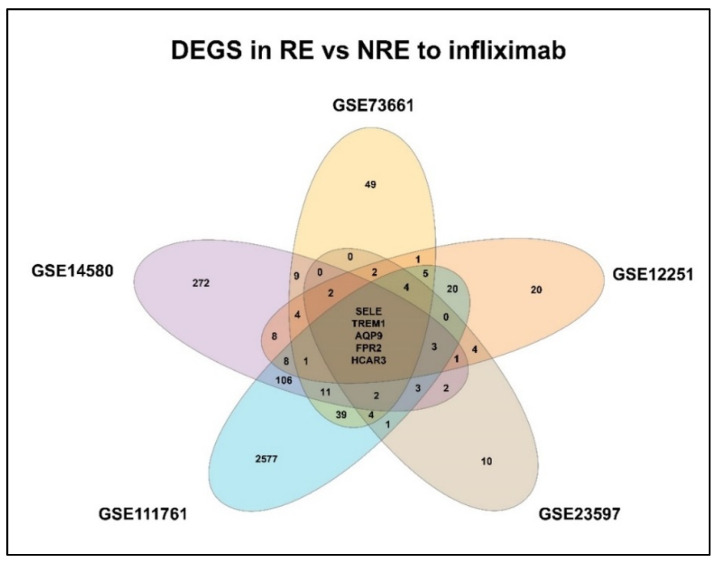
Venn diagram of identified differentially expressed genes (DEGs) shared between the datasets. RE: Responders; NRE: Non-responders.

**Figure 6:**
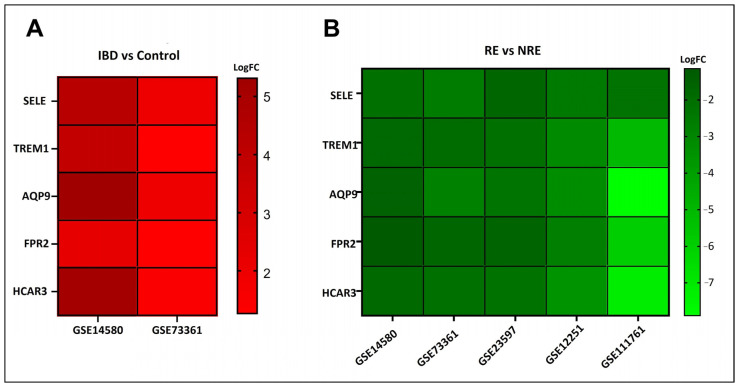
Log fold change (log FC) heatmap of differentially expressed genes (DEGs) of (**A**) IBD in GSE14580 and GSE73361, and (**B**) infliximab responder group in various datasets.

Gene expression signatures of RE and NRE were recorded in the five datasets (Supplementary Tables S4–S8, Figures S2–S6). Then, they were further divided according to whether they were upregulated or downregulated in RE (Figure 4). A further intersection was performed, and five DEGs: selectin E (SELE), aquaporin 9 (AQP9), formyl peptide receptor 2 (FPR2), triggering receptor expressed on myeloid cells 1 (TREM1), and hydroxycarboxylic acid receptor 3 (HCAR3) were common across—all five datasets (Figure 5), which might indicate their role in response to infliximab, making them interesting biomarkers for predicting treatment response. The five genes were downregulated in the infliximab responder group (Table 2 and Figure 6B). Furthermore, the five identified DEGs were among the 174 DEGs found in IBD patients compared to healthy controls and were all upregulated in the IBD patient group (Figure 6A).

### 3.3. Gene Enrichment Pathway Analysis 

To understand the pathways where all five genes are involved, the genes were uploaded to the Metascape tool. It identified three DEGs (SELE, TREM1, and FPR2) involved in the inflammatory response pathway (Table 3). 

### 3.4. The Normalized Expression Values of Five Common DEGs in Each Dataset

**Figure 7:**
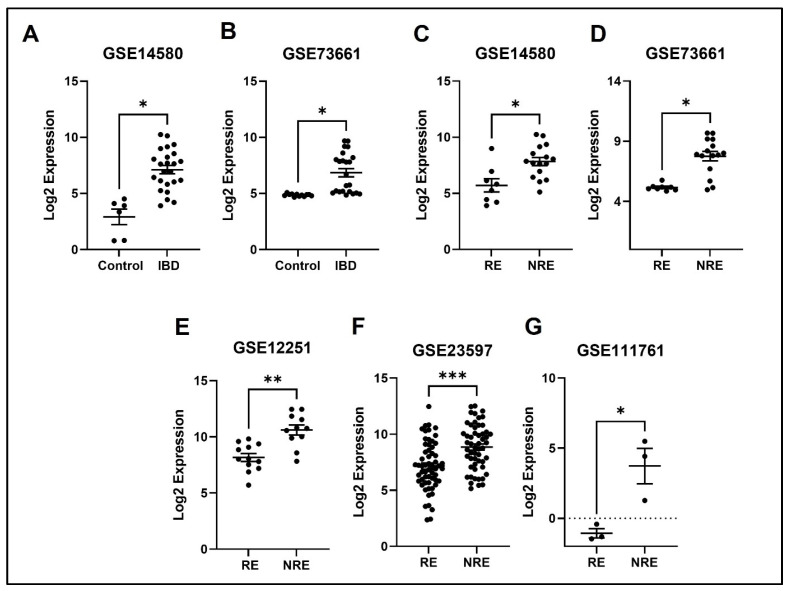
Expression of the SELE in different datasets (**A**,**B**) SELE expression increased in IBD patients compared to healthy control. (**C**–**G**) SELE was found to be downregulated in responders compared to non-responders in all five datasets (* *p* < 0.05, ** *p* < 0.01, and *** *p* < 0.001). IBD = inflammatory bowel disease, RE = responders, NRE = non-responders.

**Figure 8:**
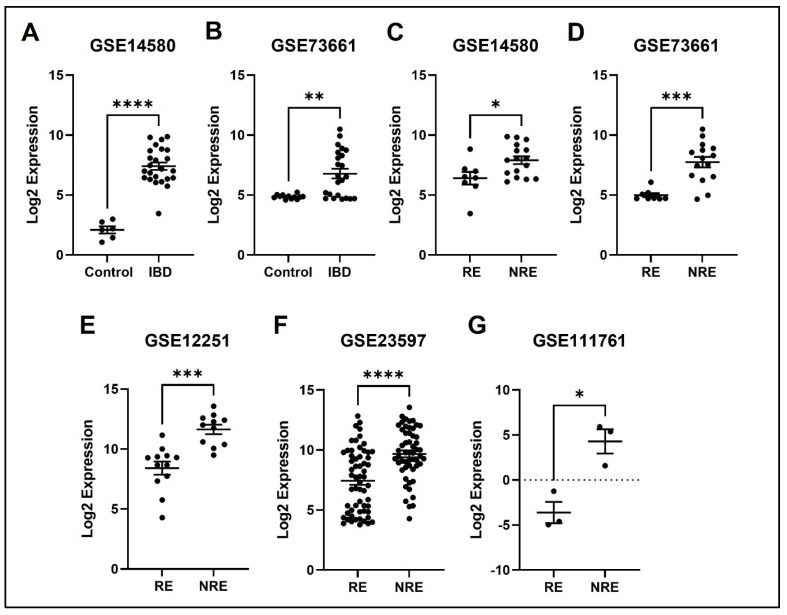
Expression of the APQ9 in different datasets. (**A**,**B**) APQ9 expression is increased in IBD patients compared to healthy control. (**C**–**G**) Decreased expression of APQ9 in patients responding to treatment compared to non-responders in all five datasets (* *p* < 0.05, ** *p* < 0.01, *** *p* < 0.001, and **** *p* < 0.0001). IBD = inflammatory bowel disease, RE = responders, NRE = non-responders.

**Figure 9:**
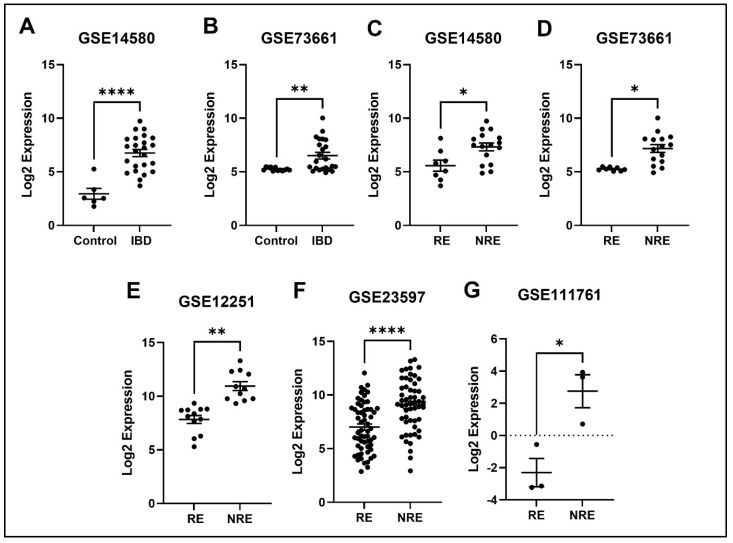
Expression of the FPR2 in different datasets. (**A**,**B**) TREM1 expression is increased in IBD patients compared to healthy control. (**C**–**G**) Decreased expression of TREM1 in patients responding to treatment compared to non-responders in all five datasets (* *p* < 0.05, ** *p* < 0.01, and ****p < 0.0001). IBD = inflammatory bowel disease, RE = responders, NRE = non-responders.

**Figure 10:**
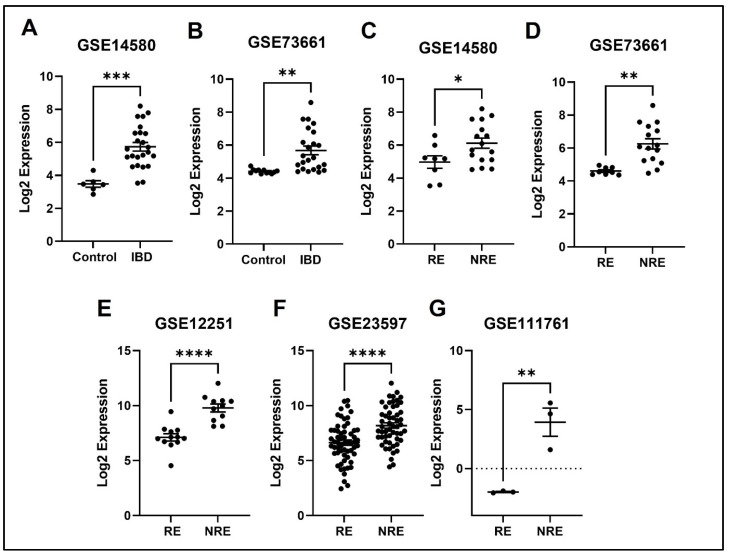
Expression of the TREM1 in different datasets. (**A**,**B**) FPR2 expression is increased in IBD patients compared to healthy control. (**C**–**G**) Decreased expression of FPR2 in patients responding to treatment compared to non-responders in all five datasets (* *p* < 0.05, ** *p* < 0.01, *** *p* < 0.001, and **** *p* < 0.0001). IBD = inflammatory bowel disease, RE = responders, NRE = non-responders.

**Figure 11:**
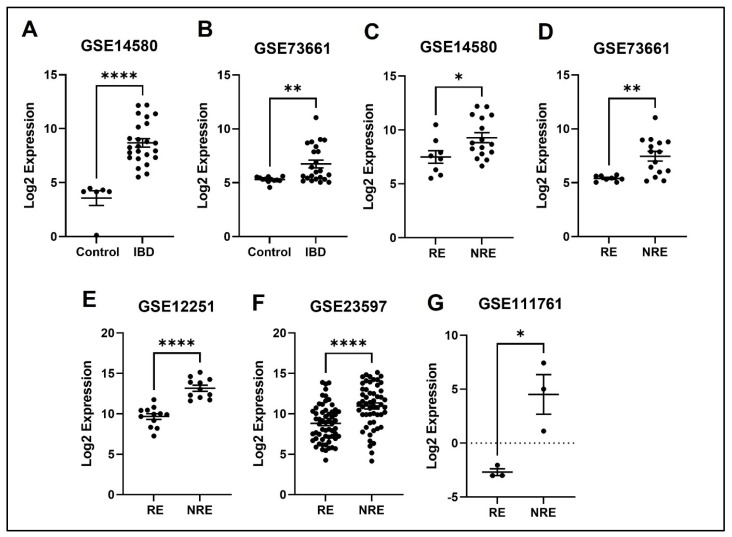
Expression of the HCAR3 in different datasets. (**A**,**B**) HCAR3 expression is increased in IBD patients compared to healthy control. (**C**–**G**) Decreased expression of HCAR3 in patients responding to treatment compared to non-responders in all five datasets (* *p* < 0.05, ** *p* < 0.01 and **** *p* < 0.0001). IBD = inflammatory bowel disease, RE = responders, NRE = non-responders.

The expression profile for each of the five identified genes was studied in healthy control samples, IBD, then RE and NRE groups. Interestingly, there is a clear differential expression pattern across the five identified genes; all these genes’ expression is significantly upregulated in IBD patients compared to healthy individuals. They are also significantly upregulated in the infliximab NRE group compared to RE (Figure 7, Figure 8, Figure 9, Figure 10 and Figure 11).

## 4. Discussion

After the detailed analysis of several datasets obtained from the GEO omnibus, five DEGs (SELE, APQ9, TERM1, FPR2, and HCAR3) were identified. The mentioned DEGs are hypothesized to be specific biomarkers involved in the pathogenesis of IBD and infliximab response. Those five genes’ expression is significantly upregulated in IBD, which implies that the over-expression of those genes plays a role in the pathology of the disease. Moreover, our findings suggest that the overexpression of those genes in the infliximab NRE group could also be why this group’s symptoms persisted and did not present a better response to treatment. Having given a background on the general findings, a summary of each of the five DEGs in the existing literature and their IBD association is presented in the discussion section. 

### 4.1. SELE 

SELE gene encodes for the protein E-selectin, which is part of the selectin family involved in the inflammatory response, particularly cytokine-stimulated response. Moreover, it is responsible for migrating leukocytes to sites of inflammation by mediating the adhesions of cells to vessel walls [22]. E-selectin is predicted in multiple cellular structures, including caveola, clathrin-coated pit, perinuclear region of cytoplasm, and vascular walls [23]. Our results indicate that patients with IBD had higher SELE expression than healthy controls. Likewise, several studies found that E-selectin expression was induced in IBD patients, specifically during inflammation; thus, it is considered a marker of inflammation severity [24,25]. Furthermore, a high level of circulating E-selectin is also correlated with other inflammatory diseases, including rheumatoid arthritis, Grave’s disease, and systemic vasculitis, indicating the importance of the SELE gene in inducing inflammation [26,27,28]. TNFα has been shown to induce the expression of various inflammatory molecules, including E-selectin [29]. Therefore, we postulate that inhibiting TNF-alpha through infliximab is supposed to decrease the expression of E-selectin, which is consistent with our findings illustrating the downregulation of the SELE gene in the infliximab response group (Figure 7). On the other hand, the SELE gene was still upregulated in the NRE group. A study comparing multiple genes expression, including SELE, through immunohistochemical staining between infliximab RE and NRE found that staining was much stronger in biopsies from patients with poor response to infliximab [30], which is consistent with our findings.

### 4.2. AQP9 

AQP is one of 13 members (AQP0-AQP12) of the aquaporins family that has been widely studied in the past few decades, and it is further sub-classified into the aqua glycoprotein subfamily [31]. They are considerably small (26–34 kD) and very hydrophobic, with intrinsic membrane proteins, and AQP9 specifically contains two additional peptide spans compared to the other subtypes. The AQPs family is found in the plasma membranes of various cells and functions to transport fluid across the cell membrane [32]. In a study that aimed to obtain and characterize a dataset of significant DEGs of treatment naïve UC patients compared to healthy controls, 1480 DEGs were found in UC patients. Interestingly, AQP9 was one of the most prominent expressed genes in this study [33]. Another study aiming to identify specific universal markers for chronic inflammation in IBD found five genes (AQP9 was one of them) significantly upregulated in the IBD group [34]. These results are consistent with our results demonstrating increased AQP9 expression in IBD patients compared to healthy control (Figure 8). The increase in expression in IBD could be in response to inflammation to restore the disturbances in the epithelial barrier of the colon, as AQP9 is found in membranes of tight junctions and plays an essential role in regulating tissue-specific physiological properties in tight junctions in ulcerative colitis [33,35]. Our analysis found that AQP9 was downregulated in infliximab RE compared to NRE. The same results were reported in a study looking at immune cell infiltration and its association with the pathogenesis of CD, using the GEO database that found three DEGs, with AQP9 amongst them, were considered diagnostic markers. Interestingly, AQP9 was found to be decreased in CD after patients underwent anti-TNF alpha therapy, and no changes were observed in the non-responder group [36]. AQP9 was reported to be most strongly expressed in cancers, including hepatocellular carcinoma [32,37]. The data suggest that it is involved in suppressing hepatocellular carcinoma invasion through HIF-1α expression in hypoxic tumor microenvironments [38]. Moreover, AQP9 expression is involved in colorectal cancer and is considered a predictive biomarker for chemotherapy response. Upregulation of AQP9 was also associated with enhanced response to chemotherapy and patients had higher rate of survival rates than those with low expression [39]. 

### 4.3. FPR2

FPR2 is a seven-transmembrane G protein-coupled receptor that modulates immune responses [40,41,42]. It is expressed in multiple cells, including epithelial and endothelial cells and most immune cells [41]. FPR2 binds to several ligands like chemotactic formyl peptide, derived from mitochondria or produced by bacteria [43]. Once activated, the receptor elicits pro-inflammatory responses and increases monocyte chemotaxis and neutrophil recruitment [44]. FPR2 has been thought to manage the primary recruitment and activation of phagocytes to bacterial and host-derived ligands, thereby contributing to colonic epithelial homeostasis, inflammation, and tumorigenesis [45]. On the other hand, it was observed that FRP2, at the later stage of inflammation, illicit anti-inflammatory effects by releasing mediators such as lipoxin A4 and resolving D1 inhibiting and damping the inflammation [41]. This highlights the importance of FPR2, acting as a key central checkpoint in inflammation. FPR2 is considered a novel gene involved in IBD’s pathogenesis, and very few studies have investigated its effects. A study involving mice tested the capacity of FPR2 in stimulating inflammatory cytokines, including TNF-α, IL-1β, and IL-6, key cytokines in IBD development. It was observed that mice that express FPR2 had increased TNF-α and IL-1β compared to the FPR2^–/–^ mice. Interestingly, the study found that TNF-α and IL-1β were also upregulated in FPR1^–/–^ mice, suggesting that the inflammatory effects were mainly due to FPR2. This validates the results of our study indicating increased expression of FPR2 in IBD patients compared to healthy controls (Figure 9), as well as its involvement in inflammatory response according to the Metascape results [45]. 

### 4.4. TREM1 

TREM1 is an important receptor found on plasma membranes of myeloid-derived cells, including neutrophils, monocytes, and macrophages [46]. It is involved in various physiological functions and is heavily implicated in the immune system. Indeed, myeloid-derived cells are antigen-presenting cells that interact with T-cells to induce oral tolerance and activate the immune response in case of an invading pathogen [47,48]. TREM1 gets activated by sensing a local stimulus that activates it, causing the secretions of pro-inflammatory cytokines and amplifying the immune response [49]. In IBD patients, TREM-1 is believed to be involved in sustaining a chronic inflammation process, and several studies have shown that it is overly expressed, especially in active areas [50]. A study analyzing the intestinal mucosal expression of TREM1 in IBD patients indicated an increase in expression in the diseased group compared with the healthy controls [51]. This is consistent with our findings showing overexpression of TREM1 in IBD patients across both datasets comparing IBD with healthy controls. Furthermore, it has been indicated that blocking the TREM1 pathway in mice, for example, significantly reduced colitis inflammation and severity [50,52]. Several studies have shown the association between TREM1 and response to the anti-TNF drug [53,54]. Our results show increased expression of TREM1 in the infliximab NRE compared to the RE (Figure 10). A study investigating the expression of TREM-1 in CD14+ monocytes concerning anti-TNF response found similar results to our study. It concluded that decreased TREM1 expression predicts a positive response to anti-TNF therapy. It was observed that monocytes with high expression of TREM1 had reduced Fcγ-receptor and autophagy pathway activity, which might contribute to the resistance to treatment [55]. 

### 4.5. HCAR3

HCAR3 is one of the three HCAR family members exclusive to humans and higher primates, found in multiple immune cells, including neutrophils, monocytes, and basophils [56]. It is a receptor for both 3-OH-octanoic acid and nicotinic acid, which function as negative feedback for adipocyte lipolysis and codes for a receptor for kynurenic acid, one of the tryptophan metabolites [56,57]. It was found to be involved in multiple diseases in humans. For example, HCAR3 is one of the DEGs involved in coronary artery disease [58]. Furthermore, its expression is increased in colorectal cancer, breast cancer, as well as cervical cancer and was negatively correlated with survival rate [59,60,61]. A study hypothesized that HCAR3 aids in the interplay between ingested and gastrointestinal microbiomes by modifying the functions of the immune system [62]. This suggests that it could be involved in inflammatory conditions, including IBD, highlighting the findings of our analysis which revealed HCAR3 to be upregulated in patients with IBD compared to healthy controls (Figure 11). In another study, A meta-analysis was conducted using publicly available transcriptomic datasets to identify metabolic pathways that might be involved in IBD pathogenesis and found that HCAR3 is upregulated in IBD patients compared to the healthy control, further consolidating the results of our study [57]. Moreover, this study also showed that expression of HCAR3 was decreased in infliximab-responders compared to non-responders, which is in line with our results. 

## 5. Conclusions and Clinical Implications

The current study used publicly available transcriptomic data to identify DEGs of importance in the pathophysiology of IBD and response to the popular anti-TNF drug, infliximab. 174 DEGs were identified across two different datasets (GSE14580 and GSE73661) and are shown to be involved in IBD. Following further analysis, five DEGs (SELE, TREM1, AQP9, FPR2, and HCAR3) were significantly upregulated in the IBD patients who do not respond to infliximab treatment, serving as potential biomarkers for infliximab resistance. Moreover, the five genes identified in this study have protein products that are secreted into the blood; this can lead to the use of non-invasive methods, such as blood testing, for monitoring the response of infliximab in IBD patients. Furthermore, due to the ease of test taking, patients can monitor their response throughout their treatment course. Patients who do not appear to be responsive to infliximab can be identified at an earlier stage and opt to change their treatment options. This will lead to a better overall prognosis for the subset of patients who are non-responsive and will help clinicians in individualizing treatment. In conclusion, these genes’ normal expression and interaction may be required for effective infliximab response and to maintain healthy gut homeostasis. Further analysis is needed, along with experimental validation, to identify the potential biomarkers that could be used to predict the infliximab response in IBD patients. Therefore, future studies investigating infliximab response and the pathophysiology of IBD should consider the impact of altered gene expression with an initial focus on the genes identified/highlighted in this study.

## Figures and Tables

**Table 1:** List of GEO datasets that met the inclusion criteria (Experimental type = Expression profiling by microarray).

Accession Number	Title	Sample Size	Organism	Sample Source	Comparisons
GSE14580	Mucosal gene signatures to predict response to infliximab in patients with ulcerative colitis	Healthy control = 6 IBD = 24 (Responders = 8 Non-responders = 16)	Homo sapiens	Colon (The biopsies were collected 15–20 cm distal from the anal verge)	IBD vs. control, Responders vs. non-responders
GSE73661 *	The effect of vedolizumab (anti-α4β7-integrin) therapy on colonic mucosal gene expression in patients with ulcerative colitis	Healthy control= 12 IBD = 23 (Responders = 8 Non-responders = 15)	Homo sapiens	Colon (Biopsies were taken in the colon at the edge of ulcers whenever present. If no ulcers were seen, then biopsies were taken at the most inflamed colon segment)	IBD vs. control, Responders vs. non-responders
GSE12251	A Predictive Response Signature to Infliximab Treatment in Ulcerative Colitis	Responders = 12 Non-responders = 11	Homo sapiens	Colon	Responders vs. non-responders
GSE23597	Expression data from colonic biopsy samples of infliximab-treated ulcerative colitis patients	Responders = 54 Non-responders = 59	Homo sapiens	Colon	Responders vs. non-responders
GSE111761	Differential expression of IBD susceptibility and IL23R-associated pathway genes during ongoing anti-TNF therapy	Responders = 3 Non-responders = 3	Homo sapiens	Intestine	Responders vs. non-responders

* Dataset included infliximab responder and non-responder groups; only infliximab-treated patient groups were analyzed in this study.

**Table 2:** Log fold change (log FC) of the significant differentially expressed genes (DEGs) in each of the five datasets. IBD = inflammatory bowel disease; RE: Responders; NRE: Non-responders.

List of DEGs	GSE14580		GSE73661		GSE12251	GSE23597	GSE111761
	IBD vs. Control	RE vs. NRE	IBD vs. Control	RE vs. NRE	RE vs. NRE	RE vs. NRE	RE vs. NRE
SELE	4.20	−2.12	1.99	−2.60	−2.45	−1.69	−4.79
TREM1	3.79	−1.75	1.26	−1.92	−3.11	−2.06	−5.06
AQP9	5.30	−1.51	1.93	−2.77	−3.22	−2.23	−7.89
FPR2	2.40	−1.59	1.27	−1.65	−2.36	−1.69	−5.92
HCAR3	5.12	−1.79	1.45	−2.07	−3.50	−2.14	−7.20

**Table 3:**
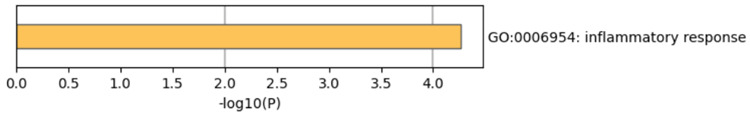
List of differentially expressed genes (DEGs) involved in inflammatory response pathway. Following gene enrichment ontology, three DEGs were considered to take part in the “inflammatory response” pathway.

Category	Term	Description	LogP	Log (q-VALUE)	Symbols
GO Biological processes	GO:0006954	inflammatory response	−4.26	0	SELE, TREM1, FPR2

## Data Availability

The datasets analyzed during the current study are available in the Gene Expression Omnibus repository, https://www.ncbi.nlm.nih.gov/geo/, accessed on 15 November 2022. Individual dataset link is provided below: Accession No. GSE14580, https://www.ncbi.nlm.nih.gov/geo/query/acc.cgi?acc=GSE14580; Accession No. GSE73661, https://www.ncbi.nlm.nih.gov/geo/query/acc.cgi?acc=GSE73661; Accession No. GSE12251, https://www.ncbi.nlm.nih.gov/geo/query/acc.cgi?acc=GSE12251; Accession No. GSE23597, https://www.ncbi.nlm.nih.gov/geo/query/acc.cgi?acc=GSE23597; Accession No. GSE111761, https://www.ncbi.nlm.nih.gov/geo/query/acc.cgi?acc=GSE111761.

## Supplementary Materials

The following supporting information can be downloaded at: https://www.mdpi.com/article/10.3390/life13030680/s1. Table S1: Criteria for selection of DEGs in this study (w/o = without); Table S2: List of genes that are differently expressed in GSE14580 and GSE73661 (IBD vs. control); Table S3: List of intersected 174 DEGs from GSE14580 and GSE73661; Table S4: List of genes that are differently expressed in GSE14580 (RE vs. NRE); Table S5: List of genes that are differently expressed in GSE73661 (RE vs. NRE); Table S6: List of genes that are differently expressed in GSE12251 (RE vs. NRE); Table S7: List of genes that are differently expressed in GSE23597 (RE vs. NRE); Table S8: List of genes that are differently expressed in GSE111761 (RE vs. NRE); Figure S1: Gene term enrichment analysis of the 174 shared DEGs of dataset GSE14580 and GSE73661; Figure S2: Gene term enrichment analysis of the 437 DEGs of dataset GSE14580 (RE vs. NRE); Figure S3: Gene term enrichment analysis of the 138 DEGs of dataset GSE73661 (RE vs. NRE); Figure S4: Gene term enrichment analysis of the 88 DEGs of dataset GSE12251 (RE vs. NRE); Figure S5: Gene term enrichment analysis of the 129 DEGs of dataset GSE23597 (RE vs. NRE); Figure S6: Gene term enrichment analysis of the 2789 DEGs of dataset GSE111761 (RE vs. NRE).
