# Supplementary material for: Identification of Specific Biomarkers and Pathways in the Treatment Response of Infliximab for Inflammatory Bowel Disease: In-Silico Analysis
Source: Life (Basel). 2023 Mar 2;13(3):680. doi: 10.3390/life13030680 (PMC10057676; doi:10.3390/life13030680)

Supplementary Material

Identification of specific biomarkers and pathways in the treatment response of infliximab for inflammatory bowel disease: *In-silico* analysis

Rachid Kaddoura[1]†, Hardik Ghelani[1]†, Fatma Alqutami[1]†, Hala Altaher[1], Mahmood Yaseen Hachim[1], Reem Kais Jan[1]*

[1] Department of Basic Science, College of Medicine, Mohammed Bin Rashid University of Medicine and Health Sciences, Dubai, UAE

*** Correspondence:**
Corresponding Author
Reem.jan@mbru.ac.ae

Table S1 Criteria for selection of DEGs in this study (w/o = without)

(A) IBD vs Control:

**Dataset**	**Criteria**	**Total number of probes**	**Total DEGs**	**Up regulated in IBD**	**Down regulated in IBD**
GSE14580	P ≤ 0.05 and logFC = ±1.5	3090	2848 (2133 w/o duplicates)	2007 (1468 w/o duplicates)	841 (670 w/o duplicates)
GSE73661	P ≤ 0.05 and logFC = ±1.5	221	213 (197 w/o duplicates)	126 (112 w/o duplicates)	87 (85 w/o duplicates)

(B) RE vs NRE: (RE= responders; NRE= non-responders)

**Dataset**	**Criteria**	**Total number of probes**	**Total DEGs**	**Up regulated in RE**	**Down regulated in RE**
GSE14580	logFC = ±1.5	586	491 (437 w/o duplicates)	98	393 (339 w/o duplicates)
GSE73661	P ≤ 0.05 and logFC = ±1.5	175	151 (138 w/o duplicates)	30 (28 w/o duplicates)	121 (110 w/out duplicates)
GSE12251	P ≤ 0.05 and logFC = ±1.5	114	110 (88 w/o duplicates)	7	103 (81 w/o duplicates)

GSE23597 (for intersection)	P ≤ 0.05 and logFC = ±1.5	50	50 (43 w/o duplicates)	8 (7 w/o duplicates)	42 (36 w/o duplicates)
GSE23597 (for Metascape analysis)	P ≤ 0.05 and logFC = ±1.5	156	148 (129 w/o duplicates)	19 (16 w/o duplicates)	129 (113 w/o duplicates)
GSE111761	P ≤ 0.05 and logFC = ±1.5	3545	3211 (2789 w/o duplicates)	1483 (1282 w/o duplicates)	1728 (1512 w/o duplicates)

Table S2: List of genes that are differently expressed in GSE14580 and GSE73661 (IBD vs control).

**Dataset**	**Condition**	**List of genes**	
**GSE14580**	**IBD**	**Upregulated (1468)**	**Downregulated (670)**
		SLC6A14, CXCL1, KYNU, DUSP4, LPCAT1, DUOXA2, DAPP1, HLA-DRA, ADAMTS9, LOC101060835, LOC100996809, HLA-DRB4, HLA-DRB3, HLA-DRB1, HLA-DQB1, ANXA1, CXCL3, LOC101060835, LOC100996809, HLA-DRB5, HLA-DRB4, HLA-DRB1, HLA-DQB1, TTC9, C2CD4A, RPS6KA2, HLA-DMA, CFB, REG4, ARNTL2, INSC, RBPMS, CASP1, CD55, TCN1, CHAC1, MMP12, HLA-DPA1, SAA2, SAA1, LOC100996809, HLA-DRB4, HLA-DRB1, CXCL2, ADM, HSP90B1, PSMB9, CD44, LPIN1, HLA-DRB1, TNIP3, S100A9, PDZK1IP1, UBE2L6, PITPNC1, DUOX2, CARD16, IRAK3, CRELD2, ARFGAP3, MIR8071-2, MIR8071-1, IGHV4-31, IGHM, IGHG2, IGHG1, DUSP6, CD40, CD74, MIR6732, ZC3H12A, GNA15, C4BPB, NOS2, SAMD9L, IL17A, ARFGAP1, SPINK4, NUCB2, SAA2-SAA4, SAA2, SAA1, MEOX1, MMP7, LRP8, IKBIP, DEFB4B, DEFB4A, ASRGL1, IDO1, BACE2, BIRC3, TRIB2, LYN, IFITM3, LOC101060835, HLA-DQB1, TIMP1, TMEM71, PI3, SLC7A11, ALOX5, VNN2, RTEL1-TNFRSF6B, TNFRSF6B, PFKFB3, PHLDA1, LYZ, KDELR3, OLFM4, TAP1,	HMGCS2, SLC22A5, SNX13, ACAT1, ACOX1, PADI2, HMGA2, C1QTNF3-AMACR, AMACR, VLDLR, SLC25A34, SLC26A2, ABCB1, APOBEC3B, PAG1, TMEM171, NLN, PPID, PCK1, SERF1B, SERF1A, RMDN2, ACADM, ZCCHC14, ABCB4, ABCB1, CNKSR3, LAMA1, USP30, CIPC, MT1F, ENTPD5, NBPF1, AQP8, EFNA1, CNNM2, CDKN2B-AS1, RHOU, PRDX6, PLPP1, MT1G, PPP2R3A, TRPM6, CYCS, DDAH2, MIER3, FRMD1, MAML3, DHRS11, ATP5S, UGT1A3, UGT1A1, UGT1A9, UGT1A5, UGT1A8, SCAPER, GUCA2A, GLS, PAQR5, SLC17A4, MT1M, SGK2, SNX30, SEMA5A, SRI, SLC30A10, NAALADL1, CDC14A, HIGD1A, LOC100996506, RETSAT,

		SLC15A3, S100A8, DPY19L1, PML, XAF1, RTEL1-TNFRSF6B, RTEL1, IFITM2, FKBP11, CDH3, NFKBIZ, ANXA5, PIM3, IFITM2, IFITM1, CDC25B, NCF4, SLA, RTEL1, SERPINA3, IFI16, UBD, GABBR1, MCUB, ASPHD2, FBXO16, MMP10, APOL1, LCN2, BAG3, CHI3L1, CCDC167, SERPINA1, ADAM15, LAMP3, SDR16C5, SERPINB5, PIKFYVE, FAM26F, KCND3, MAFF, F3, SBNO2, S100P, RRM2, LILRB2, TGM2, SRGN, TRIM69, CARD16, CASP1, SGMS1, PKIA, ERO1A, CREB3L2, IFI30, PIK3R2, GBP1, PHC1, MMP3, TAP2, MTHFD2, PCSK1, BHLHA15, QKI, CALU, STAT1, CARD6, ZBP1, CIITA, CD274, AQP9, GRK5, SOCS3, CCL24, HLA-DMB, THBD, PHLDA2, VSIG1, MLKL, RGS2, SNN, S100A2, MUC5AC, TMC5, WARS, BTN3A3, CLCN4, OSBPL3, SLC41A1, STXBP1, SERPINB7, WISP1, CXCL5, S100A11, PMEPA1, CXCL8, GLCCI1, CENPW, B2M, CLDN1, MCTP1, FCRL5, IGKC, VNN1, PKM, SLFN5, GRHL1, C8orf4, IQSEC1, PTAFR, PDE4B, IFITM1, BICD1, IGK, IGKC, IL1B, GJA4, MLIP, FAM83A, LOC100129518, SOD2, PARP8, ART3, FCGR3B, FCGR3A, OSMR, STRIP2, CXCL6, LIPG, MIR1908, FADS1, RPL22L1, DUSP7, LPGAT1, ST3GAL1, FXYD5, KLHL29, ELL2, PLAU, RAC2, NCOA7, TMEM92, PCDH17, RAB31, ASPH, AQP3, RILPL2, RHOQ, TRIM22, MME, BHLHE40, DNAJB9, RHOH, PF4, IER3, ITGB2, TMEM106A, GPX8, CEP112, AIF1, KCNE3, CD93, SEC24D, FOXQ1, ABCA12, GM2A, GJA1, ADA, TRIM29, DNAJC10, PLAUR, PLA2G7, RASGRP3, GIMAP2, HGF, BAG2, CYAT1, IGLV1-44, IGLC1, UNC5CL, MSN, REG1A, LOC101928916, NNMT, PECAM1, FFAR2, SEC14L1, TSPAN5, AGPAT4, LY96, ZAK, CASP5, LOC401317, CREB5, IGHV4-31, IGHM, IGHG3, IGHG1, IGHA1, CBR3, LYSMD2, TGFBI, PMAIP1, FAM92A1, DRAM1, DERL3, SEMA4D, LINC00888, CSF2RB, XBP1, EHBP1L1, MXRA5, IL1A, MAFB, ARHGDIB, LOC643733, MMP1, KLF7, CDC42EP2, IGLV1-44, IGLC1, PRDM1, ELOVL5,	DEFB1, WDR78, ANK3, PHLPP2, BSG, MIR4680, PDCD4, PIWIL2, FGFR2, TMEM38B, RETSAT, MT1E, RUNDC3B, ABCG2, NAAA, SETD9, APPL2, TSPAN7, GUCA2B, CDKN2B, C15orf38-AP3S2, AP3S2, LOC286052, TMEM37, NEURL1B, SLC35G1, FCGRT, ATP23, ACSF2, HSD3B2, CYP2B7P, CYP2B6, PDK4, AGFG2, METTL7B, SAMD13, LOC101929340, C2orf88, MT1H, PHYH, LEAP2, SLC4A4, EPB41L4B, RRAS2, HSD17B2, SELENBP1, RAVER2, PLA2G12B, SLC36A1, LOC100506558, MATN2, SLC38A4, PRKG2, P2RY1, DPP10-AS1, LOC102724250, LOC101930059, NBPF15, NBPF11, NBPF1, SLC51B, DEPDC7, SLC39A5, EXPH5, SYTL2, CLYBL, RAB40B, PXMP2, SLC19A3, MCOLN2, FAM162A, MPND, ADGRA3, TP53TG1, MYO1D, ADIRF, PKIB, PDLIM2, FMO5, LRRC31, LOC101927157, NIPAL1, OSBPL1A, PMM1, SLC16A1, ADH1A, TMEM56, CPT1A, TMEM98, MT2A, ZNF91, HNMT, HMOX1, PBLD, FAM47E-STBD1, FAM47E, STBD1, ARHGEF37, ETFDH, EPHX1, CES2, CLCN2, PDE6A, GALNT12, OAF, SLC20A2, LRRC19, MT1HL1, UGT2A3, MT1X, PTPRR, LRRN2, ACADS, MAOA, ABAT, PSD3, METTL7A, LOC101928274, VSIG10,

		ITGA2, TNFSF13B, TMEM163, USP18, CCL20, LST1, AZGP1, NAMPT, KLF2, CR1, RASA4B, RASA4CP, RASA4, PAM, ADAMTS1, CLDN2, MAP3K8, TSPAN11, PDP1, TAGAP, OAS2, ANKRD22, SNCAIP, IGLJ3, IGLV1-44, CKAP2, IGLV@, IGLC1, INPP5D, SELL, TRABD, REG3A, ADGRE2, MTCL1, RGCC, SLC4A11, RIPK2, STEAP4, BLOC1S5-TXNDC5, TXNDC5, C2, PLXND1, PPBP, IL7R, SHCBP1, MIR6756, MCAM, MMP19, STC1, TFF2, PMP22, CEBPB, EPHA2, EGR3, GNB5, HELZ2, MAP9, FGR, APIP, TFF1, F2R, PRDX4, LAIR2, EHD3, CHSY1, CTSH, TPK1, ROBO1, COL6A3, BLVRA, CD300A, CCL18, CKAP2, IGLV@, IGLC1, TNFAIP8, ITGAM, ACPP, ETV7, NHSL2, RNF145, THY1, MIR21, VMP1, CHST15, HS3ST1, HLA-DPB1, PROK2, COL15A1, HLA-DOA, IL1RN, TMTC1, IGKV1OR2-118, KRT6B, SH3BP5, SYTL1, FSTL1, PTPRM, CLEC4A, CXCL11, FPR1, TPRG1, COL4A1, DDIAS, SELPLG, GMFG, SFMBT2, SLCO1B3, PALM2-AKAP2, AKAP2, GRAMD2, OLFM1, BCL2A1, TMEM158, C4B_2, C4B, C4A, FCGR3B, DDX50, PTPRC, GPR65, EVI2B, KIAA0895, IGSF6, LDOC1, VWF, F2RL2, CSGALNACT1, HOMER1, DMBT1, NFIB, ELK3, CXCR2, SERPINB3, POU2AF1, GBP4, SERPING1, CKAP2L, SLAMF8, CD38, SLC9A7, TNFAIP2, KLHL5, CXCR4, RBP7, IKZF1, TNFSF8, PIK3AP1, IGHV3-23, IGHV4-31, IGHM, IGHG3, IGHG1, IGHD, IGHA1, IGH, IGHM, LINC00537, MNDA, SERPINB9, HSPA13, RNF213, SLC6A6, CASP4, LOC101928635, ALDH1A2, IGKV1OR2-108, CSF3R, LAMC2, GVINP1, NQO2, TIE1, DSG3, IGLJ3, IGLV@, IGLC1, GIMAP4, ME1, PRKCQ, Sep-06, SOCS1, FBXO6, TMEM173, C4BPA, EGR2, C1R, SELP, NCF2, RNF183, ECSCR, OAS3, CNRIP1, SHANK3, ORAI2, CLEC2B, NRAV, SLC25A37, ANKRD36, CCDC69, GRHL3, AIM2, HCAR3, ADGRG6, PLEKHO2, IRS1, IGFBP3, CLIC2, MMP9, INHBA, LOC100287413, GLYATL1, KLHDC7B, CALHM2, LRRC70, IPO11, TCF4, ADGRF5,	CA12, LOC101930053, LOC101930048, VLDLR-AS1, NHSL1, VIPR1, ITCH, SH3BGRL2, SULT1A2, VAV3, BDH1, UGT1A3, UGT1A1, UGT1A4, UGT1A9, UGT1A5, UGT1A6, UGT1A7, UGT1A8, UGT1A10, PPARGC1A, RMND5A, SLC25A23, PDK2, CWH43, FAM213A, SCIN, RBP2, LYPD8, TEN1-CDK3, CDK3, TEX11, ZNF704, NAT2, CHRNA1, ASPG, AIFM3, CNGA1, PLCE1, TUBAL3, ADH1C, ACVR1C, AQP11, CA1, SUGCT, HHLA2, ASAP3, CHN2, ACER2, CHP2, UGT1A1, UGT1A4, UGT1A9, UGT1A6, UGT1A8, UGT1A10, PRR15, SOWAHA, TRHDE, TRIM36, BMP3, PITX2, GIPC2, SLC23A1, SATB2, UNC5C, NR5A2, STAP2, PPARGC1B, MEP1A, MOCS1, LOC339166, WSCD1, CHAD, BLOC1S1-RDH5, RDH5, SATB2-AS1, SLC5A11, EDIL3, LOC101930168, LOC100509620, AQP7, ACOT11, HPGD, TMEM72, PIGZ, PRLR, LOC389332, USP2, CEACAM7, SSTR1, FDX1, AGXT, MXI1, TINAG, EDN3, SLC16A9, RPS6KA6, MXD3, NWD2, FXYD3, CLDN8, DPP10, EPB41L1, EPHX2, FAM151A, YME1L1, PPARG, LRP12, BRINP3, SEMA6A, CD177, IGSF9, ARHGAP44, SVOPL, TNNC2, LOC101927478, C17orf74, LOC101930361, LOC101929970,

		AASS, SRD5A3, IL18R1, PDE10A, ABI2, IGFBP7, IL12RB1, ISG20, SAMHD1, RPS16P5, CBL, FAM30A, TFPI, TRERF1, AAED1, CLEC7A, JAK3, PIM2, HOOK3, PXDN, PRRC2C, APOL2, AREG, CD86, TFAP2C, BCAT1, COL3A1, PPM1K, GTSE1, TMEM154, C3, FOSL1, SLC2A3, CCND2, ADGRL4, CXCL10, PLA1A, CHST11, ASAP1, FAIM2, SLC28A3, NOD2, SAMSN1, BASP1, CD37, SERPINB4, SERPINB3, MZB1, SYTL3, CDC45, HIVEP3, LOC101928173, SEC22C, IL33, HEG1, SLFN11, LUM, DSE, KLK11, TUBB6, SLCO4A1, AGT, PLA2G2A, LINC00944, THEMIS2, LEF1, FAM49A, GNB4, CFI, TFEC, STOM, CSGALNACT2, KLK10, LOC101927933, LRRC8C, HLA-DQB1, CCDC88A, IGHV4-31, IGHM, IGHG4, IGHG3, IGHG1, IGHD, IGHA2, IGHA1, BANK1, PTGS2, GAL, HSPA6, TIFA, BGN, LOC102725526, IGHV4-31, IGHM, IGHG3, IGHG2, IGHG1, IGHA2, IGHA1, IGH, RGS5, SLC6A20, IGHV3-23, IGHV4-31, IGHM, IGHG1, IGHA1, CTLA4, FBLN1, PLEKHA2, COL1A2, TNC, CKAP2, IGLC1, KCNA3, FLI1, BCL6, CD82, CEMIP, RORA, LOXL2, KCNN3, FPR3, ITPR1, BST2, CFLAR, LAX1, EIF5A2, CCR2, PMCH, CCL4, CYR61, C11orf96, LOC101927069, FGD2, ACSL4, CPVL, SIRPA, HHEX, OTUB2, NR4A2, GUCY1B3, MIR146A, FAM65B, FEZ1, SWAP70, CD53, CNOT9, GABRP, ENTPD1, FPR2, AZGP1P1, AZGP1, IL10RA, STX11, RBMS1, NUP210, TACSTD2, DUSP2, IFI44, TREM1, ANKRD36BP2, SLAMF7, IGLJ3, ICAM1, IGHV4-31, IGHM, IGHG4, IGHG3, IGHG1, IGHD, IGHA2, IGHA1, IGH, KLHL6, WDFY4, MSANTD3-TMEFF1, TMEFF1, APBB1IP, ADAM28, CYP4X1, LOC727820, EDNRA, ARHGAP9, ST3GAL5, LY9, CDH11, LINC01215, SELE, VSNL1, EGR1, SLC16A14, HLA-DOB, PRICKLE1, FZD2, SCD, RAB8B, PARVB, LAMC1, SLC9A1, C5AR1, GIMAP8, MSTO1, LINC01210, TNFSF11, SIRPB2, EAF2, MPEG1, LDB2, MUC1, CEP170P1, CEP170, SLC2A14, SLC2A3, IL1R1,	CHRFAM7A, CHRNA7, LOC101928405, MIR1247, DIO3OS, AMER3, CDHR5, PDXP, SH3BP1, PTGDR, LINC01268, SH3RF2, FAM120AOS, NAT8B, NAT8, SLC51A, CHP1, NAT8B, EIF4EBP2, TRDV3, PDZD3, HEPACAM2, TINCR, FGFR3, ZNF575, CA7, LOC727916, NOBOX, HSD11B2, MEP1B, LINC01133, SLC1A7, XPNPEP2, LDHD, HAVCR1, THRA, CNTN3, RHOV, CREB3L3, SIX5, FLJ22763, DIO3OS, SLC9A3, NDUFC1, AMN, GBA3, ANKRD9, GRAMD4, MS4A12, G6PC, ANKH, PLIN1, ABCC13, OR2H1, NXPE4, PPP1R14A, ZBTB7C, PRAP1, CAMK2N1, ZFYVE28, HOXA5, CD160, INPP5J, GSTA1, MPP7, ITPKA, FAM189A1, CELA3B, CYP2B6, CDC42BPA, SCUBE2, C1QTNF1-AS1, SLC3A1, PRSS23, NR6A1, TGM5, OR51I1, ENPP1, MBNL1-AS1, B4GALNT2, SOX9-AS1, C16orf95, PHGR1, APC, SLC26A3, LTK, A1CF, SPHK2, TARP, SPIB, CES3, BDH2, ADH6, CDHR1, LTBP3, AVIL, LOC102724612, FRAS1, PARD3, GXYLT2, BEST4, NIT1, GHR, ATP8A1, TMEM221, NVL, PBX1, ABCB11, CKB, NTRK2, ISX, OTC, ST6GAL2, CDX2, C16orf78, GCNT2, RGS3, MMP28, TADA3, WNT7B, HES5, C2orf54, ADD3-AS1, PRKAG2-AS1, SBF2-AS1, KCTD4, ZBTB10, AK5,

		PPP1R16B, DNM3, ATP11A, ARL4C, IGHV3-23, IGHV4-31, IGHM, IGHG3, IGHG1, IGHA2, IGHA1, CFAP46, MIR155, MIR155HG, EML1, MX2, PLEK, LCP2, LOC106146153, NCALD, GEM, MAPRE2, CATSPERB, TRIM40, IGLL5, CSTA, DDIT4, FYN, PDPN, FCN3, ITGA4, STK4, ENG, AP1S3, FCGR2A, CPEB4, ITGAX, LAYN, PIK3CG, RGS1, VCAN, HCLS1, IGHM, IGHG1, CXCL9, SERPINE1, LRRK2, TRAF3IP3, RFTN1, PLCL1, VAV1, HSH2D, LOC100509457, HLA-DQA2, HLA-DQA1, SP110, IFI6, RNASE6, FLJ32255, CLEC2D, TAL1, KRT7, ST8SIA4, NFATC1, IFIT3, IRF4, MUC4, ACKR4, GPR155, BTBD19, NXPE3, TMEM156, TGIF1, LRRC8E, SERPINB4, NID1, ARHGAP29, SLC5A1, NR4A3, CD300LF, TNFRSF12A, ITGA5, CCDC88B, SPARC, C1S, APOL4, DYX1C1-CCPG1, DYX1C1, KCNJ15, LOC101928361, ARHGAP15, IGHV4-31, IGHM, IGHG1, IGHA1, EVI2A, ZG16B, ICAM2, COL1A1, MCC, VCAM1, RASGRP1, SIK1, LAT2, SLC8A1, LOC103091866, LINC00623, LINC01138, LINC00869, STMN3, ANXA6, P2RY10, CD47, PRKCDBP, LTF, HAVCR2, ECT2, LINC00261, BMS1P20, RRAGD, LAPTM5, PHACTR1, FCER1G, MAP3K3, TRIM47, EMB, PLXNC1, IGK, NLRP7, ODF3B, Mar-01, IGHV3-23, IGHV4-31, IGHM, IGHG4, IGHG3, IGHG1, IGHD, IGHA2, IGHA1, IGH, CECR1, IGHV4-31, IGHM, IGHG3, IGHG1, IGHD, IGHA1, GNAI2, MADCAM1, PCOLCE, CSRP2, IGFBP5, USP34, IFI44L, PLEKHS1, FAM129A, OSM, S100A7, TNFRSF9, RASSF2, ACKR3, GLYR1, SEPT6, FOXC1, LOC100293211, ANKRD36C, KLK12, LOC101060835, LOC100996809, HLA-DRB6, HLA-DRB5, HLA-DRB4, HLA-DRB3, HLA-DRB1, HLA-DQB1, STS, CYP7B1, KLHL14, OTUD4, SUSD3, ACP5, GPR183, TMPRSS3, ZFP36L1, RUNX2, CXCL13, BNIP3, PNMA2, SNX20, PRR16, LINC-PINT, APOBEC3G, NCF1C, NCF1B, NCF1, GBP5, DOCK11, ADGRG3, TEAD4, CCL2, ODF2L, FGF2, COPZ2, MS4A4A, LINC00963, IGLL3P, CTSK, IGHV4-31,	LINC01088, LOC101928663, CDKL1, GABRA2, LOC101930458, HERC2P3, LOC101928820, FMN2, TMED6, GRIN3B, KCNE2, IYD, ADARB2, LINC00475, ARL10, LOC100506368, HNF4G, CARD10, ITGA9-AS1, LOC102724156, CA4, HSD17B3, RDH12, CYP27A1, CDRT15, VCX2, HSPB2-C11orf52, C11orf52, HSPB2, PLEKHG3, H2AFJ, ARHGAP26-AS1, IHH, KIAA1161, SUCLG2-AS1, C19orf73, LRP6, MTBP, LOC100130111, OR51B5, MAGEA1, BCL2L10, BEST2, TMEM63C, AWAT1, TRIM44, LRRC75A, CHDH, NEU3, ABCB9, PNLIPRP1, LGALS2, PPFIA3, LOC102724362, LANCL3, NOL4, EFNA5, LOC101927137, KIAA1456, GNA13, PCSK1N, ETNK1, PTPN21, PIKFYVE, RAPGEFL1, OPRM1, NR0B1, LINC01558, NR2F6, CRB1, DUSP16, TEX35, URM1, PTBP1, SYCP3, GPR26, SPAG8, SCNN1B, PXMP4, TTLL9, KRTAP4-1, DAPK2, SLC6A4, URI1, DUX4L24, DBET, LOC100291626, DUX4, LOC100288289, DUX4L2, DUX4L3, DUX4L5, DUX4L6, DUX4L7, LOC652301, DUX4L4, DUX4L8, DUX4L1, LUZP2, LOC340090, SLC17A8, GPC2, ST3GAL3, KLB, KIF26B, PTCHD4, LOC101929829, CYP2D6, CYP2D7, MAPRE3,

		IGHM, IGHG3, IGHG2, IGHG1, IGHD, IGHA2, IGHA1, IGH, TET2, ANO6, TSPYL2, COL18A1, CYTIP, FHL3, IGLJ3, CKAP2, PABPC1L, ENPP2, CD6, WNT5A, HECTD1, HSD3B7, EMCN, RNF24, GYPC, ADAMTS2, ARHGAP30, DOK3, VIM, IQCG, LINC00473, C2CD4B, CLEC14A, IL23A, FUBP3, SAA2-SAA4, SAA4, CCR6, SLC4A7, PALMD, GRIN3A, ADPRH, NR4A1, DOCK8, C2orf74, KIAA1841, HAPLN3, LOC100507516, REG1B, IGHD, ADGRE3, NCAPH, COL6A1, CMTM3, PIK3CD, AKT3, ANGPTL2, COL5A1, FAM126A, RARRES3, COL4A4, PLA2G3, MSC-AS1, TIGIT, TSPO2, IL27RA, PDLIM4, TNS4, PTP4A3, CHN1, LCK, ZNF512B, NRG1, EPHB1, CAV2, SMARCA1, CLMP, IL13RA2, MB21D1, SNX10, GPNMB, IGDCC4, CEP128, GIT2, P2RY6, SLC26A4, SELM, BET1L, ANKRD28, MCM10, CEP19, SLC16A2, CTHRC1, CCR7, UBASH3B, PAPLN, LOC100131043, PTGDS, TTC26, ITGAL, GALNT18, ARMCX2, MCEMP1, ALDOB, WAS, GNG11, TSC22D3, FILIP1L, C1orf43, ADGRL2, ANGPT2, COL6A2, LOC102723479, RASSF5, IGHV4-31, IGHM, IGHG3, IGHG1, IGHD, IGHA2, IGHA1, S100A4, MLK7-AS1, JAM2, AHNAK2, GLIPR1, SP140, ZEB2, MMP2, LPL, ADGRF1, FOS, CCDC3, UNC13D, BTK, TNFRSF17, CYSLTR1, P2RY13, MS4A1, JCHAIN, PTGES, SLAMF1, TBC1D1, CSF2RA, TLR4, FCAR, S100A12, PLEKHF1, FCGR1B, GLIS2, IGHM, IGHG1, IGHA1, PCED1B, BBS4, PCDHB16, ICAM4, CYYR1, B3GALNT1, TGFB1, G0S2, ANGPTL4, FCGR2B, APOC1, KMO, P2RY8, SPP1, HDAC7, CCL3L3, CCL3L1, CCL3, PNOC, DOCK2, TNFRSF4, OLFML2B, TNFAIP6, ADAM12, TWIST1, MS4A6A, CALCRL, TMEM45A, PREX1, ZFPM2, IL18RAP, COL5A2, FCGR2C, MYO16, ATRX, SLC23A2, ARSB, NFATC2, P2RX5, SDC3, RHOJ, CD79A, SPARCL1, GAS1, CEP135, CHST12, GJB3, FAM46C, LOXL1, HS3ST3A1, LOC101929272, FAXDC2, MGP, EP400, S1PR3, LOC340340,	NEURL2, AMACR, ARRB1, PPP2R5A, KIAA0141, HR, C2orf82, ALPPL2, ALPP, SLC13A2, LGR4, MYL1, LOC101927196, NPY1R, KRT12, BMP1, MINCR, KCNIP4, HIST1H2BJ, HIST1H2BG, METRN, SMIM1, UNC5B, CDH4, ZNF789, SLC5A10, ANGPTL1, ITGA7, GRM5, PKDCC, APBA1, PAPOLB, KCNG1, TBC1D21, RCC1L, FASTKD2, PROM2, IST1, SCTR, CACNA2D4, LOC101928000, RPF1, CYP4B1, PAX8, LOC101930243, LOC101929865, CAPN13, TUBGCP2, PCNX2, OLMALINC, PP7080, CADM4, PLXNB1, SLC30A4, CD101, SFXN4, APOH, LOC100127938, TNPO2, PLA2R1, ZFP41, GLI4, MVB12B, MFSD9, ANKRD18A, PROSER2-AS1, PCYT2, PDE7B, ZNF7, LOC100506125, C2CD4C, PARD6B, SDR42E1, CNFN, HCRT, STAB2, LOC101928554, FCN2, ALPI, NT5DC2, KRT40, SSUH2, GOLGA2P3Y, GOLGA2P2Y, SORBS1, LOC285097, LOC101929047, HERC2, LOC645321, PLG, IL6R, RECQL5, THSD4, FAM219B, C1orf21, RRN3P1, ZNF229, SMCO1, CBR3-AS1, CCDC14, EGLN1, PDE5A, LDLRAD4, PNLIPRP2, LINC01191, FOXP2, GDF5, SLCO1A2, FOXG1, CELA2A, CELA2B, FLJ35934, LOC101930067, CELA3A, NUDT14,

		ANKRD44, WIPF1, LYL1, NRP1, SDC2, PTPN14, MYO5A, COL12A1, CFP, NPHP1, FAM167B, MRAP, LOC100293211, IGHV4-31, IGHM, IGHG3, IGHG1, IGHA1, LRRC34, PKP4, PHC2, TLR8, MMP14, CHI3L2, RGS18, ADAM8, IGHG1, WDR19, CTSE, FGF7P3, FGF7P6, FGF7, HS6ST2, HELB, OSBPL10, IGLJ3, CKAP2, IGLV@, IGLC1, RAB38, RGS16, TNFRSF11B, COL28A1, CKAP2, PRKCB, EDNRB, FOSB, PDE4DIP, EPN2-IT1, EPN2, HVCN1, PTGFR, PAPPA, EMP3, FCGR1CP, FCGR1B, FCGR1A, PLK3, CR2, ZAP70, C10orf10, SH3RF3, LOC101928940, TIMP2, NLRP1, SERPINF1, TSPAN4, CRNDE, AFAP1L1, CLDN18, FYB, DUOX1, TLR2, TNFRSF10C, CDR2L, RAI2, KANSL3, CCDC150, NT5DC4, MFNG, CD48, DACT1, CD27, C1QA, ARHGAP25, DEFA5, MDFIC, CAV1, LOC101930405, IGHG1, RASGEF1B, CRTC3, SIT1, TRAC, TRAJ17, TRAV20, TRDV2, CD69, HSD11B1, DEFA1B, DEFA3, DEFA1, ZNF107, FGD5, PRRX1, SLC1A3, FBLN5, TM4SF1, PRSS54, HLA-DPB2, GZMB, PI15, PGF, FLJ10038, RUNX3, SBF1, CADM1, ZNF532, ARHGAP24, IL21R, SENCR, PITX1, LOC100128288, CLCA3P, SLC16A4, NDST2, MIR3945HG, ELL, WNT4, LRRC32, TESPA1, CD83, NFAT5, FBN1, GPSM3, CORO1A, AKR1B1, CLEC4D, LOC101927027, ATP8B2, HS3ST3B1, CCL19, SOX17, NCF1, FCN1, LTB, CD163, RGS4, CCDC78, CLEC4E, DCN, ANKDD1A, IL11, GAMT, CFHR1, CFH, FAP, TMEM119, LOC100506860, LCP1, EPHX4, NSD1, APOE, GPR84, DOCK4, LOC102724708, SPG20, BUB1, DEFA6, MS4A7, FCRL3, TFR2, RAB3IP, TNRC6C-AS1, KRT6A, DUOXA1, APCDD1, KIF3C, SERPINB1, IL10, SLC16A7, CAPN6, GCSAML, PALD1, CXCR1, DBF4B, LYPD6B, CROCCP2, HLX, EBF1, RAB39B, PARVG, LOC101929777, WNT3, PIK3R5, SGIP1, SLC17A9, GPRC5B, ZNF521, LOC101928817, S1PR2, SPAG4, KLHL41, NPSR1-AS1, LINC00173, KLHDC4, CYP1B1, CD180, APLNR, CCR10,	ZNF439, TYSND1, HPDL, SPTLC3, HHIP, ATOH7, SARDH, GDPD2, CD37, SPIN2A, C8orf46, DIO3, LOC728099, SPSB3, DACT2, CNTN4, EYA2, COMP, BCHE, LZTS1, SAP30L, LOC390705, FSIP1, MUC20, KLF3-AS1, TMEM131, KNG1, KIR3DL2, KIR3DL1, HSPB6, OSR1, SH2D6, LOC284578, TCP11L2, TGFA, LOC284570, GPR37L1, NAP1L2, BTG3, PLAC1, TMED3, ASB4, DNMBP, LOC100507283, CGB1, LHB, SNRK-AS1, KCNV1, NAALADL2, CYP3A43, LINC01006, C7orf13, CYP4F2, METAP1D, HSPB3, ZP2, GEMIN7, CPA6, Mar-03, AADACL2, LINC01351, UTF1, NCKAP5, HS6ST3, DNAH1, KLHL34, SLC25A30, OR10A4, DISP2, CADM3, ANPEP, CSRNP3, NYX, HOXA-AS2, CPEB2, NREP, PLA2G4C, HNRNPC, LOC101929109, ADAMTS9-AS1, CSHL1, COL6A1, EDA, MROH7

		FLJ30679, PCDH12, GALNT6, NPL, CLU, ZSCAN30, SNAI3, TRPS1, NOX4, TMED10, FAM219A, RHBDF2, SAV1, LOC100996782, MED18, SLC7A1, COPE, MGAT5, SMOX, GYG2, IGF2BP3, SEMG1, FAM124A, CACNA2D1, ITIH4, NRP2, RAB30, DDR2, DYRK3, LRRC25, SLAIN1, LAG3, ENO2, UBN2, GUCY1A2, SPRR1B, ITGB2-AS1, TCL1A, IL2RA, F5, KIAA1462, FADS2, LOC102723407, IGHV4-31, IGHM, IGHG4, IGHG1, IGHA2, IGHA1, CASC10, CLIP4, FST, TUSC3, FKBP10, ISL2, HBEGF, FDCSP, PLEKHG2, IL6, IKZF3, PLEKHO1, CCL22, GPC6, FCRLA, ZNF530, PCDH7, FCRL2, ABCA13, FZD10, EHD1, DKK3, FGF7P3, SLC2A6, CD80, PCDHA1, PCDHA2, PCDHA3, PCDHA4, PCDHA5, PCDHA6, PCDHA7, PCDHA8, PCDHA10, PCDHA11, PCDHA12, PCDHA13, PCDHAC1, PCDHAC2, PCDHA9, CD19, IL22, KLK7, AK7, TOX2, DPYSL3, LOC101927345, LOC101060632, LOC101059949, ANKRD20A12P, DAZL, TLR10, SYT14, FCMR, DAND5, ADAMTS3, SH2D1A, ZBED2, CMTM2, GPX7, DTX1, PLEKHB2, PHLDB2, LOC101929141, CCDC144CP, CCDC144B, CCDC144A, COL14A1, PRSS35, IGHG1, IGHD, IGHA2, IGH, MILR1, LOX, KIAA1549, MAPK11, ABTB1, CD52, HMGCLL1, ST3GAL6, LILRA2, DNAH5, KLF12, LILRB1, TDO2, DICER1, LY6E, NLRP3, FCRL1, NKAPL, DIO2, IL4I1, GUCY1A3, CD247, GREM1, ADAMTS12, GPR19, SNAI2, TCERG1L, SRGAP3, TAB3, SH3GL1P1, LINC00535, GINS4, CRB1, PPM1M, LINC00528, INSL4, GALNT15, RAD54L, SLC4A8, PRAC1, DNAAF3, MSR1, LINC01094, RCOR3, EID2B, KDM6B, XCR1, MSX2, CLEC11A, PPP2R3C, KCNAB2, PIFO, LOC441666, CRIP2, FLT3LG, SLC6A11, LOC101928565, ANK1, TIMP3, TMEFF2, LOC101930416, LOC101929792, LOC100996724, PDE4DIP, RAET1E, SORCS2, CABP7, ZNF7, POSTN, CR1L, CR1, SEL1L2, GPR37, IL10RB-AS1, NCOA6, SIGLEC1, MED1, TBC1D19, ARHGAP26, SIRPG, IGLV6-57, CARMN,	

		MIR145, IGHV4-31, IGHA1, HTR7P1, SPI1, NOVA2, CDC25C, ARSK, SLC18A1, SLC25A31, COPA, PTPRCAP, LOC286121, NMT1, MUC5B, LOC285847, MIF-AS1, CCNF, RCSD1, ZBTB24, C18orf54, ROPN1L, CMKLR1, STON1, SCARA3, FCRL4, IQGAP3, PAX5, C17orf51, WDR91, NOS3, ACAP1, MEOX2, MAST1, MPDZ, LILRA5, SNHG24, LOC339803, WWTR1, ADGRL3, FGF12, DOCK10, KRT17, JUP, ROBO2, CLECL1, STAP1, NLRC4, FAM20A, ATF6B, MEG3, BHMT2, MAP4K1, TBX6, PARP15, MCPH1, ACKR1, FERMT2, PLCXD3, PKHD1L1, MIR646HG, KIAA0754, CDKN2A, BTN2A2, PYCRL, CCK, CCDC80, GAGE3, S100B, TRPC3, NME5, PDLIM7, ZNF264, LOC102724660, EFCAB3, AICDA, ZNF568, RASSF8, LAMA2, POU2F2, GLT1D1, MYBL1, NNMT, LOC101928510, RASSF9, EGFL6, WASIR2, C4orf45, GPR82, PTPN12, P2RX7, KRT6C, KRT6B, KRT6A, SNORA11E, SNORA11D, MAGED4, MAGED4B	
**GSE73661**	**IBD**	**Upregulated (112)**	**Downregulated (87)**
		SLC6A14, C8orf4, S100P, CD55, REG4, EPHA2, C2, AREG, PHLDA1, LPCAT1, DAPP1, TRIM29, NR4A2, MUC5B, SNORA7B, PARP8, , IL1B, TIMP1, TRIM22, NR4A1, SNORD3D, SNORD3C, SNORD3B-2, SNORD3A, SNORD3B-1, ARNTL2, CDH3, PCSK1, CCL20, SLC28A3, SERPINB5, CXCL1, PI3, TFF1, OLFM4, SOCS3, VNN1, CATSPERB, FOS, CXCL2, PTCH2, SPINK4, LCN2, CYR61, IL1RN, EGR1, CFB, FOSB, APOL1, PDZK1IP1, HBEGF, ABCA12, RGS2, MIR221, KCND3, PFKFB3, TRIM40, GABRP, HLA-DRA, HLA-DQA1, UBD, DUOX2, OAS2, SLCO1B3, PTGS2, NOS2, SLC7A11, RNU5B-1, LOC102724428, SIK1, DUOXA2, ITGA5, IDO1, IRAK3, C4BPB, SLC2A3, WARS, KYNU, CLDN1, REG3A, VSIG1, SELP, OSMR, IL1A, TNC, SAA2, DEFA5, GBP4, STEAP4, CXCL11, SERPINB7, DMBT1, TCN1, MMP7, TNIP3, MMP3,	APOBEC3B, PDE6A, C1QTNF3-AMACR, AMACR, BRINP3, VLDLR, CYP2B7P, PHLPP2, NAALADL1, BMP3, BMP3, ENPP1, PRKG2, SLC22A5, SLC38A4, AQP8, PDK2, DPP10, SLC35G1, WDR78, CNTFR, TMEM63C, RMDN2, HOXA6, FGFR2, CDKN2B, RPS6KA6, SLC51B, PBLD, HSD17B2, TUBAL3, LRRC19, SLC4A10, TRHDE, TMEM37, TMEM56-RWDD3, TMEM56, CYP2B6, CNTN4, GBA3, SLC16A1, SLC17A4,

		DEFA6, TGM2, CEMIP, SELE, DSG3, C3, CFI, CXCL8, S100A8, REG1A, VNN2, CD274, MMP1, CHI3L1, AQP9, SERPINB3, PLEK, MMP12, SERPINB4, REG1B, MS4A1, CXCL10	SLC30A10, SLC10A5, PAQR5, ABCG2, GXYLT2, UGT2A3, MT1H, MT1F, SLCO4C1, EDIL3, MT1M, CNTN3, MT1G, ACSF2, DHRS11, OTOP2, SATB2-AS1, ABCB1, SLC51A, HMGCS2, PPARGC1A, CD177, HNF1A-AS1, B4GALNT2, MEP1B, SLC26A2, LINC01559, XPNPEP2, MEP1A, GUCA2B, PADI2, TRPM6, TMEM236, FSIP2, CHP2, HSD3B2, GHR, GUCA2A, TMIGD1, ANPEP, ADH1C, SLC16A9, CLDN8, CWH43, PCK1,

Table S3: List of intersected 174 DEGs from GSE14580 and GSE73661

List of intersected 174 DEGs from GSE14580 and GSE73661
SLC6A14, CXCL1, KYNU, LPCAT1, HMGCS2, DUOXA2, DAPP1, CFB, REG4, ARNTL2, CD55, SLC22A5, TCN1, MMP12, CXCL2, TNIP3, PDZK1IP1, DUOX2, IRAK3, PADI2, C1QTNF3-AMACR, AMACR, VLDLR, C4BPB, NOS2, SPINK4, MMP7, SLC26A2, IDO1, ABCB1, APOBEC3B, TIMP1, PI3, SLC7A11, VNN2, PFKFB3, PCK1, PHLDA1, OLFM4, RMDN2, S100A8, CDH3, MT1F, AQP8, MT1G, APOL1, LCN2, CHI3L1, TRPM6, SERPINB5, KCND3, DHRS11, S100P, GUCA2A, TGM2, PAQR5, SLC17A4, MT1M, MMP3, PCSK1, SLC30A10, NAALADL1, CD274, AQP9, SOCS3, VSIG1, WDR78, RGS2, PHLPP2, WARS, SERPINB7, CXCL8, CLDN1, FGFR2, VNN1, C8orf4, ABCG2, IL1B, PARP8, OSMR, GUCA2B, CDKN2B, TMEM37, SLC35G1, ACSF2, TRIM22, HSD3B2, ABCA12, MT1H, TRIM29, HSD17B2, REG1A, SLC38A4, PRKG2, SLC51B, IL1A, MMP1, CCL20, OAS2, REG3A, STEAP4, C2, EPHA2, SLC16A1, TFF1, PBLD, IL1RN, CXCL11, PDE6A, SLCO1B3, LRRC19, UGT2A3, DMBT1, SERPINB3, GBP4, DSG3, SELP, PPARGC1A, PDK2, CWH43, TUBAL3, ADH1C, AREG, CA1, C3, SLC2A3, CXCL10, SLC28A3, CHP2, TRHDE, CFI, PTGS2, TNC, CEMIP, MEP1A, CYR61, SATB2-AS1, EDIL3, NR4A2, GABRP, SELE, EGR1, SLC16A9, PLEK, CATSPERB, TRIM40, RPS6KA6, CLDN8, DPP10, SERPINB4, ITGA5, BRINP3, CD177, SLC51A, MEP1B, NR4A1, REG1B, XPNPEP2, CNTN3, GBA3, FOS, MS4A1, CYP2B6, ENPP1,

B4GALNT2, GXYLT2, GHR, FOSB, DEFA5, DEFA6, TMEM63C, HBEGF, CNTN4, MUC5B, ANPEP

Figure S1 Gene term enrichment analysis of the 174 shared DEGs of dataset GSE14580 and GSE73661

(A) Pathway analysis:

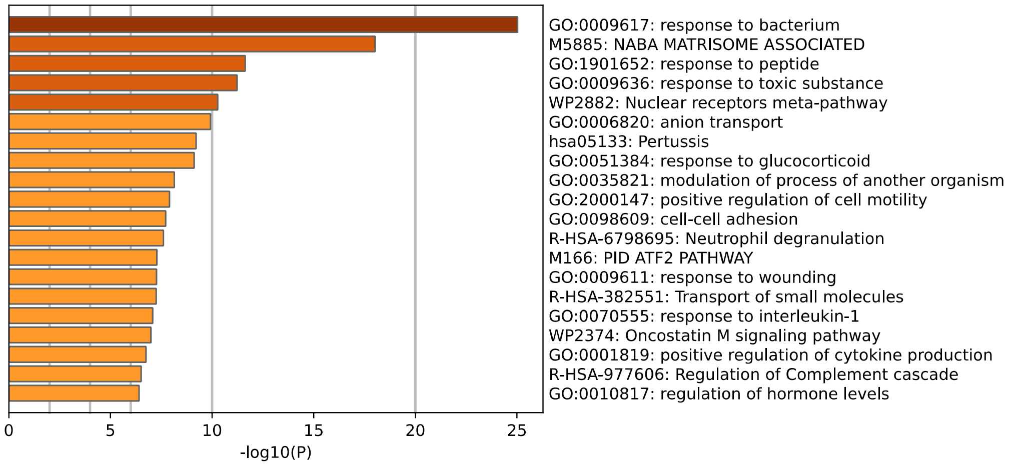

(B) Transcription factors:

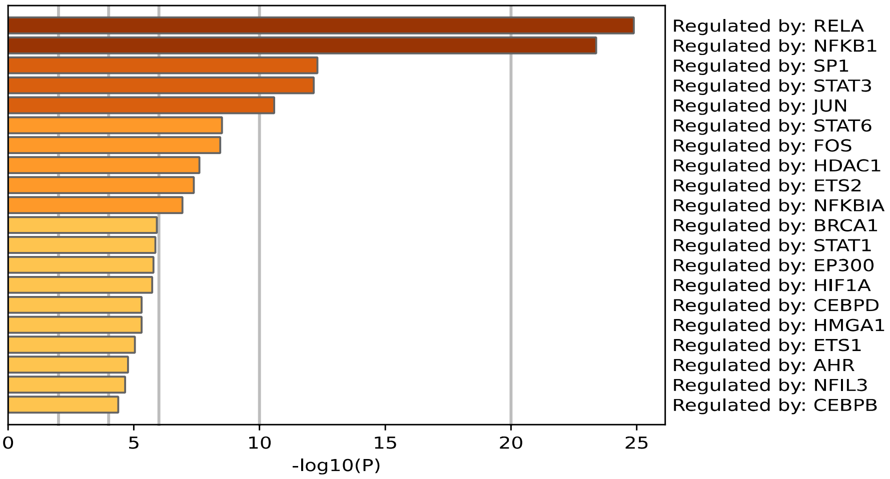

Table S4: List of genes that are differently expressed in GSE14580 (RE vs NRE)

**Dataset**	**Condition**	**List of genes (437)**	
**GSE14580**	**RE**	**Upregulated (98)**	**Downregulated (339)**

		FAM86C1, SIRT4, BBOX1, LOC105371226, DLX5, VASH1, PDZD3, SPACA9, HIST1H2BB, TAL1, GOLGA6L6, LINC01241, LOC100505824, OR10A4, GDPD2, THSD4-AS1, RPUSD3, GUCA2A, TTC34, LOC284630, DIP2B, TRIM8, CYP2C9, DKFZP434L187, NAV3, OR2B2, POU2F3, IVL, CACNB2, CNGA3, LARP4B, PCDHGA11, PCDHGC3, SH2D6, GUCA2B, LINC01029, ADGRF1, IQGAP2, SLC26A3, SVOP, C3orf62, CHP2, PDZRN3, HOXB8, GART, LINC00901, CRB1, PAX7, C9orf84, ZNF443, RECK, CHST9, CA1, KLK15, PDSS2, LINC00550, C6orf1, PNPLA1, DCST2, MIR7-3HG, ANKS1B, IDUA, HAGLR, COG6, LOC100507654, LINC00853, SLCO1C1, DCT,	HGF, PDPN, KATNAL1, IGFBP5, PDLIM4, ABTB1, PTGFR, PAPPA, ETV1, ZEB2, KLHL6, IL13RA2, IL4I1, TNC, TFPI2, NRCAM, MEIS1, AKAP12, DZIP1, IL11, MGC40069, TRAC, TRAJ17, TRAV20, TRDV2, YME1L1, ERO1B, ANGPTL2, , MAF, WNT5A, RGS5, RNF24, SPG20, RHOJ, IL7R, RASSF9, CYLD, CSF3, TMOD1, FBN1, FGF7P3, FGF7P6, FGF7, SPOCK1, TYSND1, KCNN3, TMEM207, TAGAP, XCL2, XCL1, ZNF559, IGLV6-57, IGHV4-31, IGHM, IGHG3, IGHG1, IGHD, IGHA1, SLA2, SNX29, Sep-06, C1S, CBARP, ZNF287, WTAP, RAB39B, DMC1, KIAA1462, PTGS2, C11orf96, S100A8, MMP3, CXCL6, SMIM4, CD86, STC1, IGHV4-31, IGHM, IGHG3, IGHG1, IGHD, IGHA2, IGHA1, FLT1, LILRB3, BICC1, IGHD, TXLNB, MR1, FAP, DUSP2, IGHV3-23, IGHV4-31, IGHM, IGHG1, IGHA1, SSX4B, SSX4, LILRA2, CXCL5, IL23A, TRBV19, IL6, GPR183, IL24, INHBA, ANGPT2, RGS1, AOX1, SDC2, CCL2, CLEC4E, FCGR2B, ARHGAP25, ZSCAN12, IGHV3-23, IGHV4-31, IGHM, IGHG3, IGHG1, IGHA2, IGHA1, LILRB2, IGLJ3, CKAP2, CCL8, DKK3, LOC101927933, LRRC8C, MME, TNFRSF11B, FYB, FAM49A, ZNF613, MYO1G, MMP10, CYAT1, PLAU, CCL22, LOC285957, LOC102725271, NTM, LOC101060091, KANK3, IGK, CHST11, MEOX2, RBMS1, P2RY13, LINC00888, IGKC, CALCRL, MANEA, TNFRSF4, VPS53, STEAP4, CSF2, IGLV1-44, PCBP3, MAST1, SYNE1, GLT1D1, LOC100293211, GLIS3, IGK, IGKC, SCNN1G, SLC5A12, NRP1, ZEB1, SLC6A6, CCDC88A, NRG1, DCN, MIPOL1, PRKAA2, THBD, IGHM, SLC6A15, IGLJ3, GPR37, P2RX7, SELE, SNORA11E, SNORA11D, MAGED4, MAGED4B, BRE-AS1, PMP2, JAK3,

		CDH26, C8G, GP5, CDHR5, IGHG1, KLHL20, RGL3, LINC01568, LOC643201, PDZK1, LOC102723648, DEFB105B, DEFB105A, NAALAD2, AKAP14, MIR99AHG, PSMG4, METAP2, RP1, TCF24, BEST4, RETNLB, ADAM30, LINC00427, LOC253573, NLRP2, EBF2, AQP8, LOC441666, LOC100996809, HLA-DRB5, HLA-DRB4, HLA-DRB3, HLA-DRB1, CLDN8, XIST, HLA-DQB1	MIR100HG, JAM3, HYPM, LILRA6, VCAN, TLR2, GZMK, ZNF710, DNAH1, LOC102723407, IGHV4-31, IGHM, IGHG4, IGHG1, IGHA2, IGHA1, CTB-12O2.1, S1PR3, TCF4, MECP2, IGLC1, FGF7, FZD2, PPM1K, DNAAF1, MAK, FST, CD84, S100B, ACAP1, PTHLH, PI15, COL8A1, IGHV4-31, IGHA1, DIO2, NR4A3, SAMD4A, EBF1, SERPINE1, NSUN3, ITPR1, TNFAIP6, TIMP3, DPY19L2P2, SCARA3, IGSF6, IGLL5, LOC102725526, IGHV4-31, IGHM, IGHG3, IGHG2, IGHG1, IGHA2, IGHA1, IGH, THSD7A, CCL18, GABRA2, GAS1, TEAD2, RGS4, KCNJ15, ZMYM2, TREM1, C5orf47, MCF2L2, RTN1, IGHV4-31, IGHM, IGHG1, IGHA1, BCL2A1, ERFE, CCL5, AGK, CP, FLYWCH1, IKZF3, SV2B, PTPRC, EDNRA, PROK2, MMP8, SEMA3A, CYR61, SEL1L2, GPR37, IGKV1OR2-108, RFTN2, CLMP, PKHD1L1, LILRA3, AQP9, LOC100506538, IFI44L, PACRG, PLD5, P2RY8, WNT16, MIR675, H19, PCOLCE, ZNF391, IGHV4-31, IGHM, IGHG4, IGHG3, IGHG1, IGHD, IGHA2, IGHA1, IGH, OMD, TPSAB1, SLC25A53, LINC01424, LOC780529, CYP24A1, MSX1, IGLJ3, CKAP2, IGLV@, IGLC1, MDFIC, RASSF5, ABCC9, IGLJ3, IGLV1-44, CKAP2, IGLV@, IGLC1, SH2D1A, TRPC4, ACSL4, FCRLB, CD28, FAM20A, COL12A1, RASGRF1, TRDN, FPR2, FAM126A, CDH19, SLC16A10, CHI3L1, ZNF80, MBLAC2, MNDA, EBI3, CCL3L3, CCL3L1, CCL3, FGF2, CNOT2, PNOC, PRRX1, SMARCA1, RBMS3, UMODL1, ACOD1, WDR17, PAK3, GALNT15, LOC101927482, LCA5, ADAMTS1, COL6A1, SAMSN1, CCDC141, CEMIP, CFTR, IGHM, IGHG1, IGHA1, TENM3, PARPBP, LOC101928728, TLR8, ITPR2, HCAR3, C17orf78, CXCL8, ART4, ADAMTS2, SIX4, CYP26B1, EMB,

			SLC25A30, ZNF208, PADI4, NAPSB, MYL9, LOC645485, IGHV4-31, IGHM, IGHG4, IGHG3, IGHG1, IGHD, IGHA2, IGHA1, FNDC1, AGTPBP1, IGHM, IGHG1, NKAIN2, AGTR2, ZNF826P, EBF3, GABRB1, FCRLA, RASSF8, HSF5, NAA16, GREB1, VMO1, LILRB1, ZNF678, NLGN4Y, CYP7A1, NPY2R, MCF2, RGS13, KLK7, MMP1, ASPN, ERAP1, C11orf58, KRT6C, KRT6B, KRT6A, IGHV1-69, STAP1, UGT2B17,

Figure S2 Gene term enrichment analysis of the 437 DEGs of dataset GSE14580 (RE vs NRE)

(A) Pathway analysis:

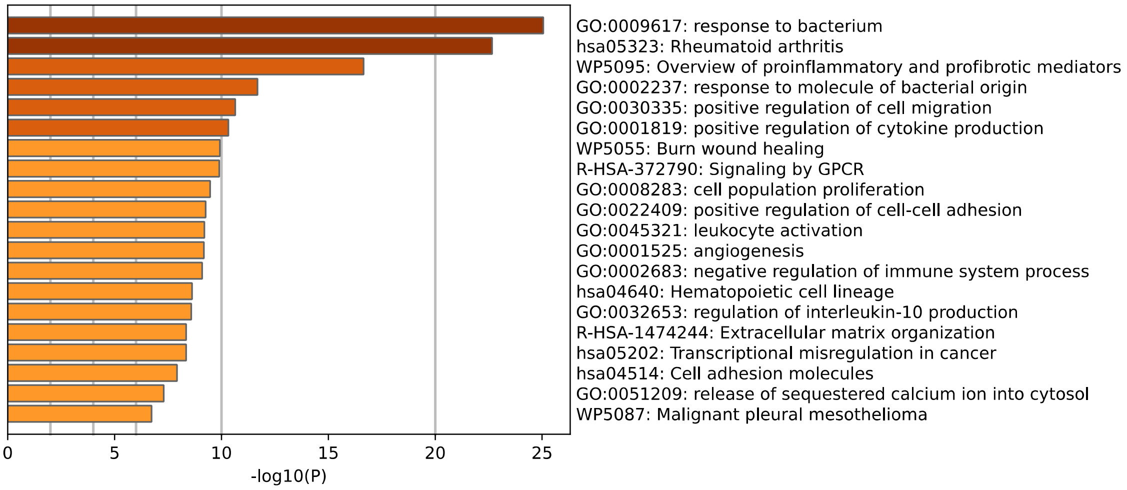

(B) Transcription factors:

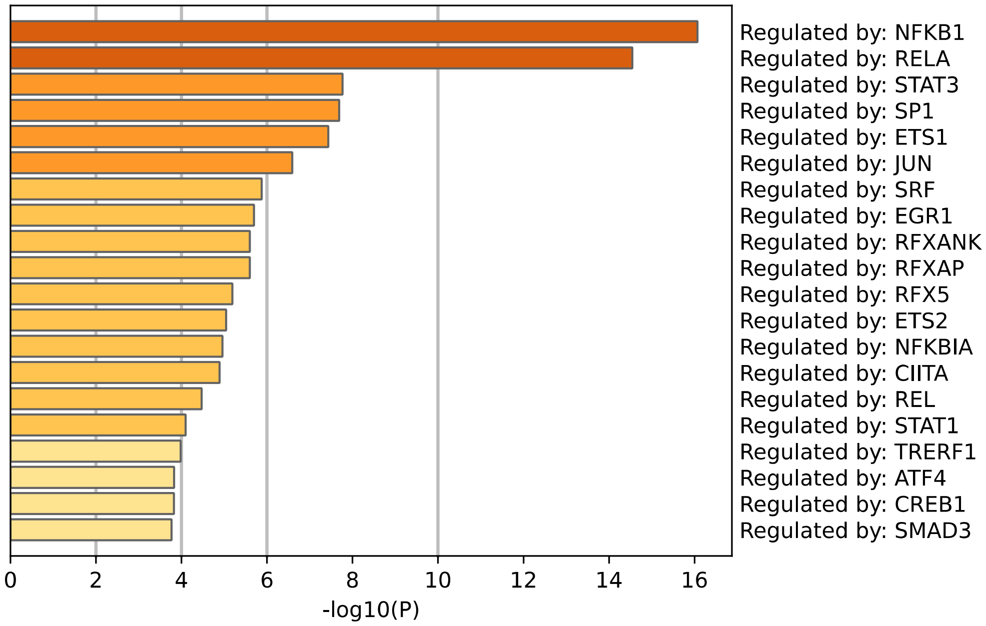

Table S5: List of genes that are differently expressed in GSE73661 (RE vs NRE)

**Dataset**	**Condition**	**List of genes (138)**	
**GSE73661**	**RE**	**Upregulated (28)**	**Downregulated (110)**
		SLC26A2, AQP8, CHP2, PADI2, UGT2A3, ABCB1, MT1M, HAVCR1, ADH1C, TMIGD1, GUCA2A, CWH43, TRPM6, GLRA2, PCK1, CPA6, BEST2, HMGCS2, GUCA2B, HEPACAM2, CAPN13, CA7, SCNN1B, CA1,	FCGR3B, FCGR3A, STC1, KYNU, IGK, IGKC, SCFV, IGHM, SCFV, IGHV3-30, SELE, , ITGAX, CXCL8, IGKC, SCFV, IGLJ3, IGHM, IGHA1, SCFV, C14orf99, IGK, IGKV3-20, IGHV3-23, IGHV3-30, IGHV3-48, IGHV3-69-1, SLC7A11, ADGRE2, IGHM, IGHV3-48, IGHV3-69-1, IGHM, IGHG1, IGHD, IGHA2, IGHA1, IGHA2, IGHA1, IGH, SCFV, IGLJ3, IGHV3-23, IGHV4-31, IGHM, IGHG2, IGHG1, IGHA1, SCFV, C14orf99, IGK, IGKV3-20, IGHV3-23, IGHV3-30, IGHV3-48, IGHV3-75, IGHV3-69-1, IGHV3OR16-7, PIM2, CSF3R, SAA2, MMP10, SCFV, IGLJ3, IGHV3-23, IGHV3-41, IGHV4-31, IGHM, IGHG2, IGHG1, IGHA1, IGH, C14orf99, IGK, IGKV3-20, IGHV3-23, IGHV3-48, IGHV3-75, IGHV3-69-1, IGHV3OR16-7, AQP9, IGLJ3, IGHV3-23, IGHV4-31, IGHM, IGHG1, IGHA1, SCFV, C14orf99, IGK, IGKV3-20, IGHV3-23,

		ZG16, OTOP2, ABCG2	IGHV3-48, IGHV3-69-1, IGHV3OR16-7, PLAU, S100A8, S100A9, SCFV, IGLJ3, IGHM, IGHG1, IGHD, C14orf99, IGHV3-48, IGHV3-69-1, IGHA1, SCFV, C14orf99, IGHV3-48, IGHV3-69-1, FCRL5, IGHV3-69-1, IGHV3OR16-7, CXCR1, CXCL1, MMP3, SLC6A14, MIR155, MIR155HG, LOC102723407, IGHV4-31, IGHM, IGHD, IGHA2, LOC102723407, SKAP2, IGHA2, IGHA1, IGH, CHI3L1, IGHV3-48, IGHV3-69-1, IGHV3OR16-7, TNFRSF17, CYTIP, SERPINE1, TNIP3, VNN2, SRGN, FPR1, FCGR1CP, FCGR1B, FCGR1A, LAX1, NCF2, RAB31, SLAMF7, SAA1, CXCR2, MNDA, IL1B, STEAP4, PLEK, MMP12, FPR2, ABCA12, TREM1, PLA2G7, CCL4L1, CCL4L2, CCL4, CCL4L1, ACSL4, SELP, CD274, GBP5, VSIG1, ANXA1, IL6, SERPINB3, MMP1, FCGR2A, SERPINB4, OSM, PTGS2, CEMIP, FCGR2C, FCGR2B, OSMR, IL13RA2, JCHAIN, CCL2, CCL2, SLC26A4, NOS2, TNFAIP6, MMP9, HCAR3, EVI2B, MMP7, SERPINB7, S100A12, CCL18, TCN1, DUOXA2, SERPINA3, INHBA, INHBA, CXCL9, ACKR4, CD180, LY96, CD79A, IDO1, IGSF6, IGFBP5, TNC, C4BPA, SELL, DSG3, LCN2, REG1B, CXCL10, CXCL13

Figure S3 Gene term enrichment analysis of the 138 DEGs of dataset GSE73661 (RE vs NRE)

(A) Pathway analysis:

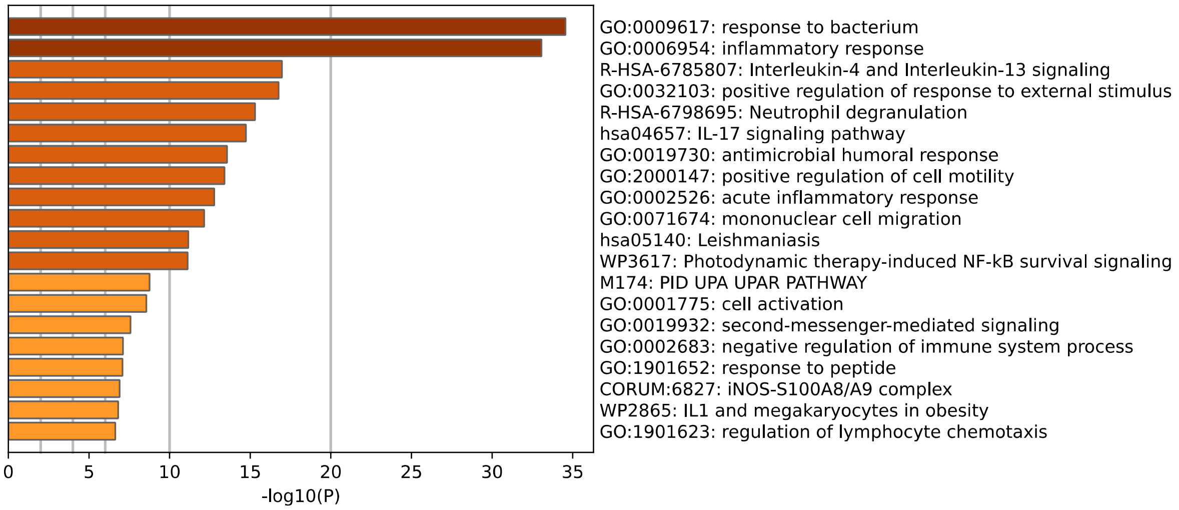

(B) Transcription factors:

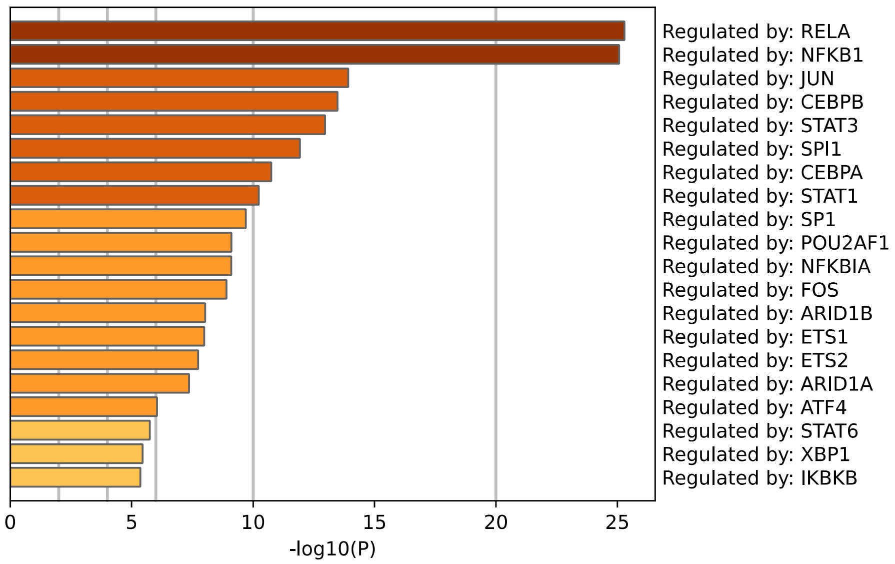

Table S6: List of genes that are differently expressed in GSE12251 (RE vs NRE)

Dataset	Condition	List of genes (88)	
**GSE12251**	**RE**	**Upregulated (7)**	**Downregulated (81)**
		WNK2, CNTN3, ZBED3, HAPLN1, DYRK1A, PCDH20, HLA-DRB4	G0S2, , HCAR3, LILRA3, TLR2, STC1, KCNJ15, IL1B, FPR2, APOBEC3A_B, APOBEC3A, IL1RN, PTGS2, TNFAIP6, TREM1, TNFRSF11B, CREB5, CSF3R, BCL6, NAMPT, C5AR1, NKAPL, ACSL1, CEMIP, FCGR1B, OSM, FPR1, ARL11, CXCR1, CMTM2, OLR1, SRGN, CXCL8, IL11, FCGR3B, FCGR3A, PROK2, LOC100129518, SOD2, TNFRSF10C, FCN1, CXCL11, PI15, TAGAP, PLEK, ADGRG3, PAPPA, STEAP4, PDE4B, DGAT2, MNDA, BCL2A1, GLT1D1, LILRA5, AQP9, FCGR3B, LOC106146153, FCGR1CP, FCGR1B, FCGR1A, MGAM, S100A12, CYP4F3, FFAR2, CXCR2, CLEC7A, IFIT2, LOC401317, CREB5, INHBA, WNT5A, LILRA2, TFPI2, SIRPB1, LILRB2, CLEC4D, FCGR2C, CLEC4E, IL18RAP, SELE, ACOD1, IL1A, GPR84, HGF, SLC1A3, NCF2, MME, EREG

Figure S4 Gene term enrichment analysis of the 88 DEGs of dataset GSE12251 (RE vs NRE)

(A) Pathway analysis:

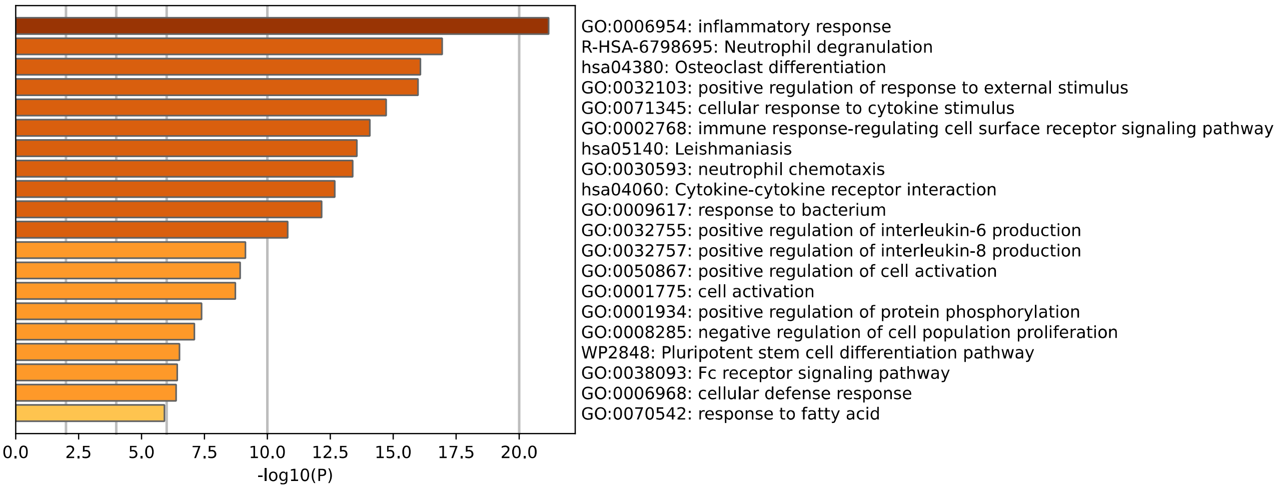

(B) Transcription factors:

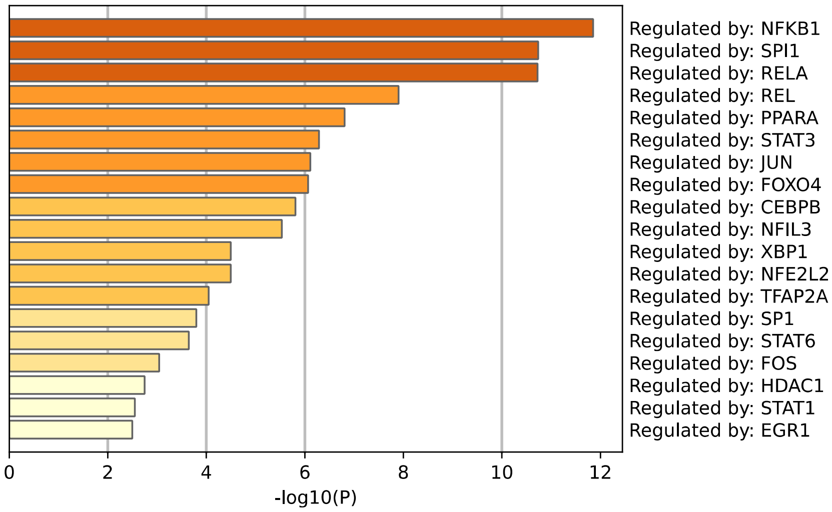

Table S7: List of genes that are differently expressed in GSE23597 (RE vs NRE)

Dataset	Condition	List of genes (129)	
**GSE23597**	**RE**	**Upregulated (16)**	**Downregulated (113)**
		LOC101929148, TTTY14, MCMDC2, FAM189A1, C2orf82, EIF1AY, HLA-DQA1, UGT2A3, WSCD1, NTRK2,	CXCL5, CXCR2, HLA-DQB1, LOC106146153, SRGN, NAMPT, TAGAP, FCGR3B, RGS5, FFAR2, CSF3R, AQP9, LOC101060835, HLA-DQB1, SPP1, LYZ, S100A8, FPR1, LOC401317, CREB5, LOC100509457, HLA-DQA1, FPR2, TREM1, CLEC7A, CDC42EP2, MNDA, CHI3L1, CCDC3, ADGRF5, FCGR2A, KCNJ15, HCAR3, OSM, TNFAIP6, C4B_2, C4B, C4A, CXCR1, FCGR3B, FCGR3A, IGHV4-31, IGHM, IGHG4,

		TXLNGY, C7orf57, USP9Y, RPS4Y1, DDX3Y, CNTN3	IGHG3, IGHG1, IGHD, IGHA2, IGHA1, IGHM, FAP, S100A9, NCF2, KYNU, COL8A1, , CTHRC1, IGHM, IGHG1, IGHA1, IL1B, CXCL1, CMTM2, SAA2-SAA4, SAA2, SAA1, SELE, LCN2, CXCL8, G0S2, VNN2, LPL, PRRX1, NLRC4, HGF, INHBA, TNIP3, MGAM, BCL2A1, ADAMTS2, CLEC4E, IGHM, IGHG1, LRRC25, PROK2, MSX2, PHACTR1, SAA2, SAA1, TNFRSF10C, GAS1, LOC100129518, SOD2, MZB1, TGM2, IGLV1-44, VNN1, VNN3, DUOXA2, FCGR2C, SAMD9L, LILRB2, IGHV4-31, IGHM, IGHG4, IGHG3, IGHG1, IGHD, IGHA2, IGHA1, IGH, COL7A1, CCL3L3, CCL3L1, CCL3, ABCA12, IL1RN, IGFBP5, CCDC88A, GBP5, IGK, IGKC, TCN1, ADGRF1, CSF3, IL1A, SERPINE1, CXCL6, STS, FGF7, GJA4, FAM30A, IGHV4-31, IGHA1, IGLV6-57, FYB, PLEK, MMP3, PTGS2, PDPN, XIST, CD300A, IL24, TLR8, REG1A, IL13RA2

Figure S5 Gene term enrichment analysis of the 129 DEGs of dataset GSE23597 (RE vs NRE)

(A) Pathway analysis:

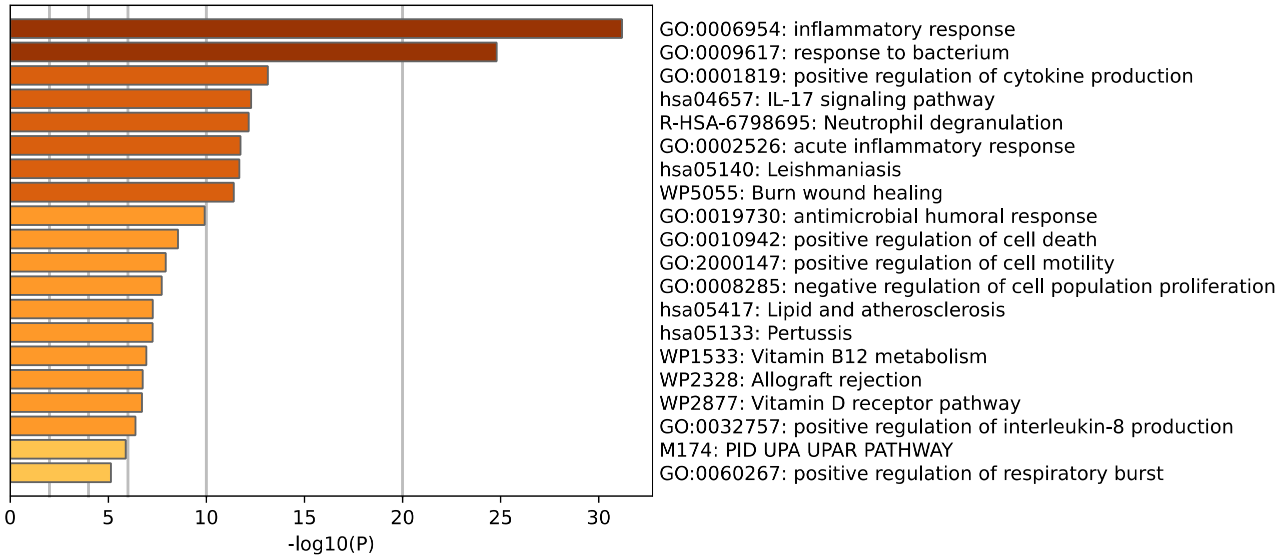

(B) Transcription factors:

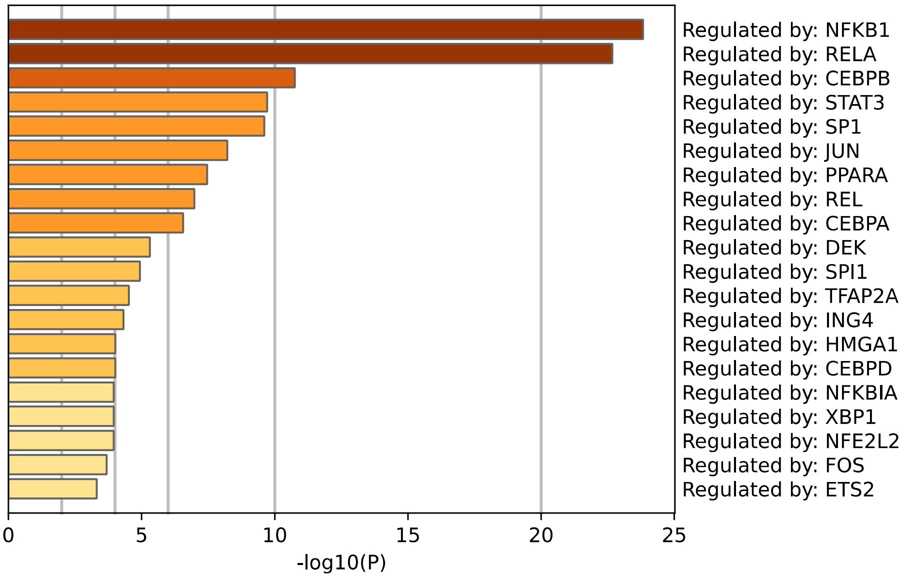

Table S8: List of genes that are differently expressed in GSE111761 (RE vs NRE)

Dataset	Condition	List of genes (2789)	
GSE111761	RE	**Upregulated (1282)**	**Downregulated (1512)**
		PLCD3, KLK1, KCNV1, CNTNAP2, LOC145837, PLEKHH1, BAIAP2L2, MYO1A, METTL7B, SGK2, FGFR3, F2RL1, C14orf176, SEMA4G, ENDOD1, CLDN8, BEST4, TMEM184A, PCSK6, FAAH, WSCD1, KBTBD11, GDA, ATG4D, LOC643650, SERF1B, GPD2, AKR1B10, PTPN20B, MALL, LRRC1, ANKS4B, THRB, RPRML, ARHGAP32, C19orf33, ACOT11, GNA11, SERF1A, GPR153, C11orf86, DHCR24, TACC2, ASL, FOXP2, VILL, GPR125, FAM47E, FLNB, CYCS, GFPT1, SVOP, C19orf21, LOC389332, GPR35, C11orf52, FLJ35024, TCF7L2, ACOX2, CBLC, ADAP1, FGF9, DHRS4L1, SERINC2, AKR7L, KAZALD1, ENTPD8,	SELL, LOC100129322, TDO2, S1PR4, FAM65B, CD70, , IGF2BP3, LOC151009, LYG2, LOC283710, LOC728684, IL17C, PGA3, SLAMF6, ZNF80, SSX3, NPBWR1, LOC100288292, RP11-165H20.1, TNXB, KLHL5, FAM153A, TSPAN32, KYNU, ZBED2, OR2T1, BCAT1, CTSL1P2, LOC150622, AIM2, FAM55C, FCRL5, RAB39B, COL27A1, ZBTB32, TLR9, TTLL4, CTLA4, TNIP3, CCDC129, STMN1, TRIM27, PTPRC, CR1, ACOXL, OLFML2A, FAM159A, RRNAD1, HAPLN3, TTYH1, UBD, PHOSPHO1, CPNE5, KCNA2, ELOVL2, CXCL1, FBXO2, TRIM53P, POU2F2, EMB, ADA, GLCCI1, SP140, ORAI2, AAK1, LAX1, FLJ40039, WNT2, CFB, FYN,

		GOLT1A, PYGB, TRIM36, HAVCR1, PTPRK, CAMK2N1, GRTP1, NUDT12, PRELID2, GJB1, LEKR1, KLB, LIMA1, FGFBP1, RAPGEFL1, C20orf54, SDCBP2, AKR1B15, LGALSL, MMP15, MAGIX, ETFB, PAPSS2, LCE3B, C20orf118, TMEM97, ABCB1, MACROD2, PAQR5, OTOP2, F12, SCGN, CGN, CELA3B, ABCD3, NPY1R, AGPAT2, SST, ZNF575, HIGD1A, LETM1, CCNI2, NR5A2, CCDC85C, SH3D19, HOOK1, MYZAP, SLC25A34, ABHD11, SPR, SPINT2, RIMKLA, PTP4A1, ATPBD4, TMEM98, GSTA2, P2RY1, UGDH, SLC16A5, ZG16, PTPRN2, AKAP1, CCDC113, SLC16A9, FSIP2, FUT5, SLC38A4, MAMDC2, SYTL2, PEX11A, FAM59A, C17orf76, KLK15, GIPC2, FAM189A1, HRCT1, C1orf226, MARVELD2, HMGA2, PLAC2, C1orf210, GUCA2A, HES5, EPB41L1, AVPI1, TYRP1, LOC100130916, TBX10, SLC25A24, PGAP3, C8orf55, GPA33, EXPH5, MUC2, TJP3, YIF1B, NGEF, MYO1D, KIAA0284, CA7, AP1M2, GLYCTK, SPRYD7, SPINT1, OPHN1, VIPR1, CYP2J2, SCGB2A1, MACROD1, TMEM56, MAGI3, ELMO3, DDAH1, COL4A6, FABP1, TTR, POF1B, MGC34796, EPS8L2, NUDT8, NR2F6, SDR42E1, SHROOM2, NOSTRIN, IL22RA1, TAX1BP3, C21orf71, CDH1, C8orf83, CMTM4, AIMP2, PRSS3, RAP1GAP, FASN, PLEKHA5,	PLXDC1, CLIP4, CHST11, HPX-2, ENTPD1, FRG2C, JAK3, CRTAC1, SOCS1, RNF170, CR1L, ODZ4, IDO1, SNX20, TBC1D3, SLC2A6, OR1S2, WIPF1, EPHB6, MORC1, SLA, TRIB2, BATF, TARP, PPP1R18, TNFRSF8, LRRK2, SERPINE1, TMEM154, RHBDF2, ALOXE3, C16orf54, ALPK2, C9orf96, ITGAX, SLC6A6, RAB7L1, FOLH1B, RASSF5, FAM113B, IL15RA, PLEKHG2, BHLHE22, ZNF714, FAM126A, PTGFR, TNFRSF9, ADORA2A, GMFG, TXNDC11, RHOH, TAGAP, SH2D1A, PIM2, KRT73, SMR3A, CD28, FMNL1, OR1S1, C1R, BASP1, CTSK, ST6GAL1, MGC16025, C20orf201, TSC22D3, PNOC, MAG, CXCR4, GSC, TXLNB, CHRDL2, CYTH4, RAC2, CDK14, INSL3, LILRB1, RIMBP3, ANKRD55, HIVEP3, MR1, FLJ32255, CRABP2, RGS16, LRRC16B, IKBIP, ARHGDIB, FAM92B, TMEM40, VAV1, CAMK1G, LOC338739, HAP1, IFI16, BNC1, TTC22, FCAR, GUCY1B3, ARHGAP25, GUCY1A3, IL6, TRIM46, CD5, KIAA0125, NOX4, FLJ41200, BVES, FSCN1, SERPING1, CORT, FCRLB, LOC284757, IL21, RASSF2, TGM2, KCNN3, ERC2, ACTR3BP5, EVI2A, LATS2, ADAMTS10, CXCL2, C5orf39, RASGRP2, TBC1D10C, DENND1C, NXPH4, TNF, FAM65C, NLRP1, FAM172BP, GPR132, FOXP3, EVI2B, CEP170, CMTM7, CD40, FBXO43, EGFEM1P, XBP1, BGN, LOC497256, CD53, PLEKHO1, FMNL3, AHSA2, KMO, CYTIP, PTP4A3, ICOS, CD79A, ANGPTL6, IL21R,

		UGT8, KIF13B, CMBL, LRRC66, FAR2, ND2, TMEM164, KIAA1671, CES2, APOH, TMEM30B, TUBAL3, CACNB2, RBP2, PTPRF, UQCR10, KRT8, GP2, NEDD4L, CPA6, VIL1, TTC38, PRR15L, TUBB4B, KRT8P12, PIP5K1B, PRKACB, LOC80054, COL4A5, SAMD13, VSTM2A, EPS8L3, PARD3, PCK1, RAB19, AKR7A3, CHST5, AIM1L, OSTBETA, COBL, PCDH20, MBOAT2, GSTA5, C2orf82, TMEM52, MESP2, B4GALNT3, PNPLA4, CFL1, DMRTA1, GOT1, MMEL1, GPR39, FAM86FP, ITPKA, CAPN2, CYP2C18, ABP1, IL20RA, CYP1A2, PPAPDC2, DGAT1, PSMG4, EPB49, CLYBL, DTX4, TMTC4, FRRS1, CDC42BPG, DNAH6, ND4, HMGCS2, MYO7B, F11R, HEPACAM2, CLDN7, C6orf136, SHROOM3, MPV17L, LOC643714, DAGLA, DNAJC11, TMEM53, ZNF704, C3orf70, COMTD1, LOC440335, SSTR1, S100A14, RDH13, VSIG2, PCDH1, CEBPG, KLK3, DCXR, NHSL1, MRPL12, TOB1, HNF4A, FAM108C1, PERP, TPMT, ATP1A4, C14orf128, C9orf7, TONSL, MPL, DHRS11, KIAA0664, MPST, LOC389023, ZNF703, C1orf201, PPARG, C10orf47, SRI, IGF2BP2, CA1, KRT83, GBA3, PANK3, IMPA2, HAS3, ARHGEF35, CHCHD10, TXNDC17, TM4SF5, DOK4, GSTO2, PCSK5, TRPM4, FAM150B, AGMAT, TTC12, SLITRK6, GOLIM4, EGFR, TST,	COL11A2, PSTPIP1, PFKFB3, RSPO1, P2RY8, PDE4DIP, MMP19, PDCD1, FUT7, NYX, IFNAR2, SLC1A3, LOC730227, CDYL2, FGR, SMAP2, TRAF1, SRGAP2, CCDC69, C11orf20, GYPC, DOCK8, TRAF3IP3, ESR1, SIT1, CADM1, MRAS, LY6E, CCR7, C14orf165, C6orf126, CHST6, ELOVL5, NTNG2, IKZF3, TNFAIP2, SLC16A11, FLI1, SLC25A45, POPDC2, LAT, OR3A2, NPY2R, LBH, BTN2A2, CSTA, KIF3C, RHEBL1, IL1A, FXYD5, TLR6, PPAPDC3, LOC100129196, IGF2, DRAM1, TNFAIP8, SLAMF1, DDIT4L, PIK3AP1, ZNF876P, CHN1, HOXC4, APBB1IP, FAP, SNURF, C1S, GVINP1, MZB1, DOK3, MEF2B, FAM89B, GPSM3, DYRK3, LOC100129648, PRDM15, R3HDML, ACAP1, FAM49A, VNN2, C9orf173, ARID5A, ZNF512B, FBXO10, TXNDC5, MAK, RASAL3, FMO6P, PARVG, SNX10, LRRC8C, BAG2, IL16, TRIM67, BCL6, LOC400958, CD180, SAMD14, ST3GAL2, PTPN7, CCDC136, BCORP1, APOBEC3G, CIITA, MTMR9LP, THY1, SH2B2, RUNX3, PPM1K, CXorf21, PIK3CD, DOCK11, ANKRD36B, KLHL6, QKI, FCHSD2, NFATC1, MEGF6, C4orf44, PRDM1, ARID3A, WAS, TRPS1, NEFH, HRH4, LOC440297, GPR65, SPTBN4, CD38, IFFO1, MYO1G, SAMSN1, LCP1, LOXL1, LOC648987, ARRDC5, BTG2, DNAJC5G, WNK4, GLT1D1, PHACTR1, ZC3H12A, BCL2A1, CNGB1, ISLR, C8orf66, CD52, PLCG2, CRYBA4, RBMY1B,

		ACTN4, TMEM177, GPRIN2, CDKL1, PTPRH, PP14571, ZNF774, PDE3A, KIAA1244, MIER3, DAOA, BTNL3, DLG3, HSD17B2, TMEM35, BDH1, FOXA3, STARD10, LOC100129516, ARHGEF10L, FH, L2HGDH, ARL4A, WFIKKN1, SLC9A1, ACR, PRSS2, ESRP1, TMEM144, BZW2, CLCN2, PLCE1, LSR, PPFIA3, LOC729680, ZBTB7C, FSIP1, DCTPP1, SLC39A5, CA2, RPS26, RBM47, CNNM4, NRG4, LOC645586, PXMP2, LOC100133311, PDHA1, TUBB8, C1orf21, DCDC2, CDH17, FZD5, GATA6, SLC44A1, C2orf72, C17orf110, TOM1L2, PRR15, FEV, CANT1, SERINC5, TAS2R39, CORO2A, ATP1A1, NRARP, CHP2, TMC4, SORBS2, ACTL8, UNC5C, ABCC3, C10orf116, PPP1R1C, EDN3, RSG1, TPRN, HNF4G, SYTL4, LGR4, DDC, MYL12B, MYO5B, CAST, TTLL12, LGALS4, GPD1L, FAM83F, SPDEF, PTPN3, GIPC1, BEND3, ZNF462, RDH5, OR52A1, SPHK2, FER1L6, PCYT2, GSS, SPPL2B, PRODH, SLC35D1, HHIP, FAM162A, CAPN1, ABHD12B, ENTPD5, ANKRD20A9P, SIAE, SULT1A4, ATP5G1, KCNG3, PRLR, ZNF497, A1CF, CLDN23, C1orf53, MGST2, ZNF326, ACOT7, TMEM141, PPP1R13B, HDAC11, PKP3, PHB, MAOA, EPS8, CBLN4, ODF3L2, CAMSAP3, C5orf32, MAPK3, MAPK13, CDS1, CPT1A, KCTD9, DBNDD2, AKR7A2P1, LOC100507410,	ARHGAP30, SLC26A11, FCGR3A, LOC100505585, FGF1, PYHIN1, ACTR3BP2, SEC14L1, TRO, SGTB, CYP26B1, TNFRSF4, P2RX7, CUBN, TUBB4A, TRIM22, LRRC34, MEX3D, FGD3, BATF3, MAPK11, TBC1D2B, LTBP2, REXO1, HIST1H1A, STRA6, CXCR2, FKBP11, FAM90A10, CARD6, TCF4, ZAP70, IGDCC4, C1QTNF3, ITPRIP, LOC100130811, SLC1A2, HOXB4, ZBP1, DCN, PIK3CG, EVC2, TREML2, CALHM2, HDAC7, RASGRP1, CEP128, RSPO3, CD3E, RILPL2, GBP5, PDE9A, PLA2G4C, GPR183, DERL3, EID3, ADAM23, NFKB2, APOL3, C11orf21, CACNA1E, DNAJB13, KLHDC7B, TNFSF13B, SERPINB9, LOC401463, CMTM3, IQSEC1, CSGALNACT2, TCL1B, LOC100653245, CCDC80, CERS4, SEMA7A, LOC652119, FAM125A, RELT, MYOG, CCL18, SPAG4, DAPP1, PLEKHO2, SLC12A5, CYSLTR1, ATP2B4, SYT11, PILRA, FBXO44, C1orf216, RUNX1, LIX1L, CASQ1, TMEM71, ARHGAP9, PPM1M, CCDC152, FYB, LOC285286, LOC100289580, IGFN1, SPRNP1, LUM, KCNAB2, ATXN2L, THAP2, LAPTM5, LINGO3, TNFRSF18, LMNB1, OAS2, LOC100133286, ALOX5, C12orf35, KRTAP4-12, LCK, LYPD1, EP400NL, FAM167B, BTK, FAM21C, SP110, IKZF1, RARA, KIRREL3, SPOCK2, TTBK1, LAT2, RINL, LOC100132790, MAP4K4, EHD3, A1BG, GHSR, RECK, FAM13C, SP140L, IL23A,

		MKRN7P, MRRF, ANKRD9, MLLT3, MIPEP, PFKFB2, NDOR1, GPHN, ALDH7A1, GRB14, SLC25A10, PTK6, SUCLG2, SCIN, FBXL22, ATP5D, B3GALT1, GALNT7, FKBP4, PADI2, C2orf89, ARHGEF26, PIWIL2, AGFG2, COX2, TRIM50, FAM55B, NRAP, ABHD11-AS1, PLEKHG3, DLD, PCCA, C9orf167, PVRL3, PPIA, PLXNA2, ZNF607, NR1H4, MICALCL, CYC1, MGST3, CTSL2, MANSC1, FABP2, NEUROD1, PHGR1, SLC20A2, OSGIN1, KCNK5, CYP2C9, GSN, PODXL2, TTPA, RAB40C, NMNAT1, ARHGEF5, CDHR5, MTUS1, ZNF681, SLC9A2, NDUFA10, KLHL32, LONRF3, UGT2B10, FLJ31104, ATP5B, ACSS2, SSH3, REEP6, PKDREJ, CYP51A1, C3orf25, SYDE2, PCDH19, FSTL4, B3GALT5, STK39, CA13, FCGBP, PITPNM3, GSR, AHCYL2, AP3S2, NBEAL1, BCAR1, RASEF, UNC13B, DOLPP1, CTNND1, SCN4B, DKFZP564C152, PDSS1, SH3RF1, C8orf38, COX6C, ND4L, PRRG2, GDPD3, SEMA6A, TLE2, GPR37L1, AQP11, COL17A1, TNNC2, TPM1, NR1I2, ALDH3A2, PLS1, KIAA1324, DHRS4, SLC3A1, KLF4, CLRN3, FARP2, CALML4, COX1, ELF3, SLC22A23, SLC39A4, UGT1A6, CHPT1, CTTN, SLC17A4, ZNF57, THNSL1, ZP3, GAS2L3, TOMM40, DSC2, BOLA3, DOPEY2, NIPAL1, SLC2A13, UGT2B11, FOXD2, C10orf108, PHYH, RIMS4, PLA2G4F, FAM83G,	H1FX-AS1, SNORA71C, RAB8B, C17orf72, MYF6, LOC100506676, ODF3B, IL10RA, PLAGL2, SLFN11, PTPRCAP, LAMP3, PGF, IL28RA, TPTE2P6, DUSP10, DTNB, TAS1R3, VIM, ACSL1, JAZF1, IGFLR1, OSM, TRPV2, IPCEF1, TFEC, C6orf145, DEF6, PRKCH, SAMD9L, SRGN, CHST15, PARP8, HLA-DOB, YPEL3, ARL11, EGFL6, PPP1R16B, HS3ST3B1, H2BFXP, CCR8, MX2, AGAP2, LOC729175, CD72, ACCN4, MIAT, EAF2, TMC8, EBI3, HR, CRYGA, PTK7, MICB, DAB1, IFI6, SELP, HECW2, LIMD2, BPIFB2, MURC, CARD8, RABGAP1, ADPRH, ELFN2, APH1B, PION, CD6, KCNJ8, TPST2, HHIPL1, MGC23284, PTAFR, HHEX, OR4X2, C9orf174, PACS1, SH2D3C, ERCC2, ZNF671, STMN3, PRDM8, PECAM1, RAVER1, ANXA6, NKX2-3, HAVCR2, ATM, DFNB59, ATMIN, PRRX1, SH3BP5, CSF3, CEP19, BMP1, NOG, C13orf15, ELMO1, LOC100129973, SLC2A14, TRIM78P, PML, TM6SF1, C1orf228, LOC645431, CENPP, CFI, TMEM156, SLC39A6, ELK3, MFNG, GNAI2, GPR133, FAM78A, SUCNR1, VSIG7, PLCB2, DFNA5, PRB3, NGF, GPR119, PARP15, FLJ42875, RAPH1, PARVB, SOX5, LPXN, CXCL3, TIAM2, GALNT14, SRRM3, IQSEC3, ADAM19, FPR2, TIGIT, LOC646513, PREX1, SLC34A1, BLVRA, SASH3, CCL4, ANKRD13D, CD3D, EID2B, LOC400743, FMR1-AS1, CYP7B1, OR51G2, TMCC2, PLA2G4E, PKP1, ARID5B, NAALADL2, CD300C,

		LOC100134229, NAPRT1, ATP6, MEST, MS4A8B, KRT20, AKAP5, MRPS35, ROCK2, LDLR, ACVR2A, OTC, HES1, ANKRD43, CYP4F12, C19orf73, PKIB, CKMT1A, PLEKHA7, CAPN5, ATOH1, RTKN, CAPN8, C5orf63, REPS2, DHRS7C, APLP2, SPRYD4, FRK, DHCR7, PGM1, RALGDS, RIPK4, RPS6KA6, KLF3, IGSF9, ABCA8, KBTBD12, COPG2IT1, SCO1, TUFT1, LRRC19, PRDX6, FAM195A, CDKN2B, EFR3B, SYT13, ZNF219, LPIN3, LUZP2, CEBPA, GPC4, INF2, PPARGC1B, PTPN21, CRYL1, FAM83B, CENPV, GPR98, COX3, LINC00483, DST, HES2, TMIGD1, LPP, CHMP2B, MYH7B, FAM171A1, SLC2A8, SLC39A11, IL17RE, ND3, LOC284578, SPTLC3, LOC285758, DTWD2, STAP2, TJP2, ECHS1, SLC25A13, DDR1, ACO2, SLC22A5, FAM101A, MVP, ALDH1L1, OR7C2, TMEM45B, MBOAT1, LOC400768, PLEKHJ1, DHRS9, PPP2R3A, C10orf58, FAM160A1, ZSWIM5, COQ3, PYY2, GGCT, WASL, ETHE1, CLMN, CASZ1, SATB2, SLC7A4, CD9, C9orf125, ATP13A4, CEACAM1, CHRM1, PPP1R36, ANKRD57, C1orf172, SH3BGRL2, CRB3, SPTBN1, FAM84B, DAB2IP, ATP7B, CORO1B, SOX6, TMEM171, APOBEC1, AQP8, ESRP2, RNPEP, SIPA1L3, ASAP2, LAMA4, CYP1A1, HDGF, HMGCS1, FAM86B1,	KIF25, RNF166, RRP7B, CD300A, C10orf10, PDE4B, HCST, ITGAL, IL2RA, LY96, IDO2, ITPRIPL1, SARDH, NAPSA, CHI3L1, APOL4, BNIP2, OR13A1, RAB31, NNMT, ASAP1, S100A4, ADAM12, Sep-01, SLC24A4, NFKBID, CSGALNACT1, DCX, WDFY4, WSCD2, GBP4, AGRP, COLQ, RNF144B, HLA-DRA, FAIM3, C8orf80, MXRA5, RORA, MYO5A, FGD2, SNN, BST2, CD84, TNFRSF6B, SELPLG, ITK, CXCR1, LOC399715, IRF5, CARD17, SLFN12, CHST2, LOC154761, Sep-06, MARCO, CSF2RB, PCOLCE, PQLC3, LOC150185, CPT1C, CMTM2, LYN, NKX6-3, CCDC88A, LOC284751, COCH, SBNO2, RNF213, ADARB2-AS1, HSH2D, DOCK2, PROK2, ITGA4, UNQ6494, POU2AF1, TBX21, SIX1, TNFRSF13C, ADAM8, S1PR3, Mar-01, RAB3IL1, TGFB3, ST8SIA4, POMC, CPXM1, ANK1, FAM20A, GZMK, SH3TC1, CD37, LSP1, LTB, LAG3, KCTD7, CD274, ST3GAL1, ZYG11A, SLC38A10, PLXNC1, STAT5A, KISS1R, RHOJ, CASP10, NHLRC4, LOC100129292, ZNF382, LILRA3, APOL2, SERPINI1, LEF1, ADD2, LDLRAD2, ZEB1, LOC100129113, EPHB1, SCARA3, CORO1A, CD2, S1PR5, ACSS3, DIO2, LOC729305, TGIF2LY, LOC284561, ARHGAP15, HAS1, CSRP2, PATL2, DLG4, KLHL17, MSL3, C17orf96, RAMP3, TSPAN11, TRAT1, CELF4, FBXO41, CD3G, AKAP2, FNDC9, SYNE1, ITGAM, IL4I1, P2RX5, SIX4,

		PWWP2B, SLC29A2, HIBCH, IFT172, SULT1A2, TUBA1C, LOC100127909, RHPN2, DENND2A, RETSAT, MTCH2, ADORA2B, UGT2B7, UNG, ATP5G3, MOB3B, MTMR8, PDP2, ZNF823, MCU, WLS, ARL14, AK2, LOC401127, SLC10A5, LRFN2, JUP, UOX, MAP7, XK, FAM84A, C5orf30, C8orf47, SHD, GMNN, PRSS8, TFF3, CAMK2D, FABP5, LOC727916, OVOL2, ESRRA, TTC19, TCF21, TMEM86B, FRMD3, CYP3A4, PLP1, IDH3A, SDSL, HOXB7, DISP2, ERBB2, GUCA2B, C14orf129, SLC4A4, HMGCR, PCK2, PKDCC, SLC27A4, NKAIN2, UGT2B15, PARD6B, OCLN, ATP8B1, HNF1A, MNX1, ELOVL6, GGT6, INADL, CHKA, WNK4, CLCA1, INCA1, GRID2IP, MGC12982, TMEM125, AMACR, EPB41L4B, TINAG, MTMR11, BCL10, CPT2, B3GNT3, STX19, CMAS, C5orf52, DYRK2, KRT18, C7orf10, ADH1C, KIF16B, B4GALNT2, ZDHHC3, FAM83H, EFNB1, ENPP1, SFN, SLC35A3, ANK3, LOC157860, GRHL2, BTBD3, CDKN2B-AS, TNXB, SYP, TRIM3, SSFA2, EPHA10, UQCRC2, LOC100133306, HOXD1, GREM2, MPP7, SEMA3B, PDZD3, CAPN13, TMEM63C, HSPB1, ARHGEF16, COQ9, GALNT12, HDHD3, NCAM1, DSP, CC2D1A, LINC00238, FUT4, FAT1, ADPRHL1, LOC100506253, ND1, CYP4F8, TNFRSF21, FAM155B, LANCL2, SPIRE2,	BMP8A, IL8, C5AR1, GRASP, MYH3, LILRB4, SCGB1A1, KCNK15, PLAU, SLCO5A1, CD48, CLSTN3, SLC17A9, C1orf190, LOC401847, STARD3, COL12A1, FER1L4, ACAN, AMDHD1, LOC100130345, LEPRE1, ANKRD23, VASH2, RGS19, ARHGEF6, E2F5, CNTNAP5, CASP5, TESC, ARSF, AQP9, XAF1, LOC100132966, SULF1, BANK1, LILRB3, RCSD1, TRPV4, DPYSL3, DDRGK1, MIXL1, MYCBP2, HCLS1, SIPA1, ADM, RASSF4, FCRL2, C16orf74, PLA1A, SAMD4A, TAF5L, SIRPG, ZNF831, CNIH3, LAMB1, LRRC15, IL26, CST7, KCNK17, HIP1, N4BP2L2, P2RY10, ACSM2B, IFITM2, TNFSF4, SLC22A15, MOP-1, IL1R1, TIFA, CD27, DKFZp686M1136, ASMT, LOC96610, LOC100128591, GPR174, LPCAT1, CAMP, CTSH, IL27RA, IFI27L2, MB21D1, PTHLH, IFI30, LAIR2, LOC100505719, WDTC1, C1orf162, ZCCHC11, CPZ, FAM46C, CCDC102A, GZMB, C1orf38, AUTS2, FILIP1L, GRAMD1A, SORCS2, OLFM1, LOC153684, INHBA, SGCD, LOC100128675, SCPEP1, MIR155HG, STAR, TP53INP1, SIGLEC9, VAMP5, S1PR2, OLFML2B, CHI3L2, CADPS2, IL12B, GATA3, LILRA6, FADS1, STAT1, ADAD1, LRRC33, HCG4, STAG3L2, PODNL1, CTHRC1, CCL25, LILRA4, IRF4, LOC285957, CECR6, UMOD, SIGLEC14, ITPR1, STAT4, FLJ43663, LMO2, MBD1, EVL, ANTXR1, TTTY13, PLEK, CBLN3, GLT8D2, IL7R, INPP5D,

		EIF6, SIRT6, SPINK2, NUDT15, CDH16, PVR, OTUD7A, HNF1A-AS1, FNIP2, LOC100129869, AGPAT9, CCDC56, MUC5B, NAT2, CTDSPL, FAM5C, RETNLB, CYP2S1, DPF3, FGFR2, GAL3ST1, DSG2, FAM110C, SPEF2, TMPRSS4, PDCD6IP, GRIA4, ACY1, PAX6, LOC729966, ZNF816-ZNF321P, MEP1A, CGB2, FRMD5, SLC16A1, FAM190A, PDGFA, DHDDS, TCEA3, PLA2G12A, CROT, ZNF664-FAM101A, COX5B, THSD4, BEND7, MVK, LDHC, FAM135B, FABP4, SNX7, ENTPD2, EMP1, C8G, PEX26, TOX3, NETO2, KALRN, LENG9, FAM81A, PPP1R16A, GEMIN8P4, CDX1, HR, NET1, MSMO1, ZIC4, LLGL2, B3GNT8, FA2H, KLHL34, MYO15B, RAVER2, MECOM, C12orf28, OVOL1, THRA, MAL2, LOC100131094, RAB27B, HADHA, KIAA2022, GNG12, IFT74, NID2, TRAP1, LIPH, CHDH, USH1C, AFG3L2, RSPO2, TMEM61, HEPH, PTGDR2, RXFP4, CHGB, SPATA2, MANEAL, TTC39A, TSPAN3, GUCY2C, DHRS1, ARHGAP8, USP43, NOL6, PTCD3, SLC30A10, APBA1, PLEKHG6, HOXA10, VDR, SLC44A3, FLJ30901, ACADS, ENHO, ADAMTS17, LINC00302, PTCH1, LOC100289255, GJB2, SAMD12, LAD1, TFCP2L1, IRF6, C9orf24, CNKSR3, CNNM2, MAPRE3, CDHR2, SLC25A5, KRTAP3-3, BDH2, ABCB11, CREB3L4, BMP3, LOC283713, LRFN4, PRSS1,	NFAM1, ARL9, CLEC1A, LINC00256A, CXCL5, ACRC, MEX3B, PACSIN1, RPS16P5, BCL2L11, C20orf106, CXCR3, RLTPR, ANKRD44, KRT81, UBASH3A, ATP6V1E2, LTA, LMO4, Sep-02, LOC729867, MUCL1, C2orf27A, GRINA, PVRIG, LOC100133669, LOC729799, GRASPOS, ELL2, C17orf87, LOC390940, AKNA, GNA15, TAL1, NFE2, MAP4K1, MECP2, ARL4C, GAB3, KCNJ15, SLC12A6, CDH6, IRF1, SH3KBP1, ANGPTL2, CD69, CARD11, CA11, DUSP4, OR10H2, GBP1, SNX29, NR4A3, CXCR6, LEPREL2, PTPRU, GPR137B, FLJ38576, IPW, MAP3K8, ICAM1, SFMBT2, MSN, CTSL1, SYTL1, KCNAB3, IFNG, LYG1, NOD2, SLAMF7, N4BP2L1, BNIP3, LY86, MFAP2, LOC100131043, C16orf5, SLC2A3, GPR19, EBF1, QPCT, LOC646743, WISP1, FLJ31713, GPX5, CD83, GRAP, C19orf59, CASP4, GCGR, TMEM132A, LY9, BLK, ATG16L2, CACNB4, FMO1, RBM14, HLA-DMA, SEMA4D, NCR3, IFITM4P, BHLHE40, SPON2, IFITM1, CELA2B, SPACA3, LILRA2, DENND3, CCR4, RETN, RBMS1, SLC43A3, BCL2L15, HELB, RHOQ, C8orf60, RFTN2, MEI1, GJD3, GIMAP4, MEF2C, EVC, COL4A4, LSAMP, PCBP3, HCAR3, UPB1, APBA2, PAPLN, KIRREL2, REEP1, TMEM108, CLEC2D, KLF7, C20orf46, LINC00426, GBP2, VNN1, COL22A1, BTBD11, ADAMTS14, CLLU1OS, VWCE, FCN3, RAB24, CXCL6, UBTD1, AGBL4, GNG7, CD19, GRHL3, CLECL1, RGS1, PKHD1L1, TH,

		MLXIP, C5orf58, LNX2, VSIG10, ANKH, PPM1L, COX5A, KRT18P55, NDFIP2, ALDH3A1, PEX11G, SPRY2, DQX1, PHLPP1, BCAS1, SNX30, MOGAT2, LOC91948, PREP, C20orf112, PPP1R14C, GALE, MYRIP, RIOK3, SUCLG1, PPP1R9A, PEBP4, MAST2, GLOD5, FAHD1, ABCC6, XYLB, KIAA0485, LOC100128498, TMLHE, CLDN4, SH3RF2, LPHN3, TOM1L1, HOOK2, CYCSP52, REP15, MYOCD, INPP5J, EDIL3, LOC100289186, CCDC64B, HTR3C, NOS1AP, ANG, CARD10, LOC100288884, ERMP1, PPP1R1B, SNAR-A3, NDRG2, PDE9A, ISX, PAWR, SELENBP1, SLMO2, RCOR2, UQCRC1, KCNK10, SMPD3, FBLIM1, PTPRR, SYT8, C18orf18, FXYD3, CGREF1, LPCAT4, C9orf152, RHOV, KIAA1958, ZNF702P, FLJ35946, PCLO, MYH11, TSPAN15, EPHA1, KIAA1919, DKC1, MS4A10, LRPPRC, GCNT2, KIAA1522, LOC100289178, LOC201651, ERBB3, DEPDC4, CYP4F2, KRT19P2, ANO10, CRYBA2, LDHD, SAG, ITGA3, KRT19, KRTCAP3, TSPAN8, SLC22A18, BRP44L, HOXA7, PLEKHA6, BEST2, GNE, ITGB4, AIFM3, EMP2, EPPK1, DNAJC22, BCL2L15, MARVELD3, C3orf52, NDNF, ADH1A, SNCB, MUC12, MGST1, OGN, HSD11B2, SEMA6D, SCUBE2, AMT, BCKDHB, SOSTDC1, C1orf106, PPP1R14D, KITLG, SHANK2, C6orf105, SHBG, DNMBP, ADCY6, GHITM,	GAS1, COL24A1, GJA4, ARHGEF3, SLC15A3, OSBPL3, GIMAP7, ATP8A2, LOC374443, GIMAP5, S100A9, RARB, CCL2, DUSP5P, C9orf30-TMEFF1, CDH26, CLEC4D, NCKAP1L, PBXIP1, RFX5, LOC100127983, LILRA5, LCP2, FCGR2A, LOC100128670, KCNE4, TMEM204, STK17B, LOC440896, UXT, C16orf86, UBE2L6, ENOX1, SOAT2, F5, CXCL10, SIRPD, DSE, TREM1, C20orf107, NUP210, CD80, ZNF469, CD82, LOC153577, STX11, RAB33A, MCTP1, TNFAIP3, PDCD1LG2, LOC254896, PLEKHF1, FNDC1, DENND5B, SLC4A3, KHDC1, FAM117A, CEACAM19, ADAMTSL4, HBG1, GIMAP6, FOXD1, FLJ45445, CLEC17A, A1BG-AS1, SLC41A1, PNRC1, DPEP2, OBSCN, FERMT3, PLGLB1, FLT3, S100A8, GNGT2, LGI2, DNAJC4, OR8J1, FLRT2, RNF24, SELE, TMEM163, CD79B, FCRL1, IL1B, C19orf66, SNCAIP, NCF4, DENND5A, PDPN, CLEC7A, TPST1, APOC1, HLA-DRB3, CXCL11, MME, ANKRD36BP2, IRAK2, GPR124, HLA-DPB1, FSTL1, KIF26B, MMP25, PKD1L2, DPYSL4, ZNF154, GREM1, GGT5, LOC100653210, GNB5, RFTN1, SLAMF8, ARNTL2, FAM167A, DZIP1L, HSPB8, C20orf195, FAM115C, MAGEC2, CCL17, PBX4, FCGR2B, ZBTB1, POPDC3, RIC3, GPR116, TXNDC3, DBNL, LOC148696, SLC16A4, S100A12, GPC3, MARK4, PROKR2, IGSF6, ALPK3, PMCH, MEGF11, SPI1, LOC100506342, GHRL, LRRC14B, CYBB, VAMP1,

		UGT2A3, PROM2, ETFDH, FUT2, WDR78, DEPDC7, PCSK9, LOC401022, LGALS3, CHP, PAQR8, BPNT1, MGLL, PLCB3, SMAGP, CLIC5, EPN3, CKMT2, NR3C2, UPK3A, BTNL8, IGSF3, CNTN3, C5orf35, EHF, NLN, FAM83E, ME3, FDFT1, GPT, AK1, GPRC5C, RAB25, GLP2R, B3GNT6, CIDEC, TMEM54, GDPD2, RERGL, PKP2, TLCD2, SPA17, DUSP8, FLJ32063, KIAA1161, PRIMA1, VSX1, C1QTNF9B-AS1, MPI, CLTB, GABRB2, ARSD, NMNAT3, ST14, KCNIP4, ADAMDEC1, KIAA0895, MEP1B, SPDYE2, LRRC16A, RNF152, TMEM139, SUV420H2, MFSD4, BLVRB, FAM55D, KIAA1239, KCNK1, ABO, SLC25A1, HSPB3, GOLM1, TSPAN1, SLC35D3, TRIM7, MUC20, KLC4, PA2G4, IHH, EPB41L5, MYOT, GPRC5A, FOXA1, LOC723809, CD2AP, TMEM100, RHOF, RASSF7, HOXA13, ZDHHC23, GGT8P, GALNT3, UGT1A8, ACAT2, PRR5L, BMPER, MAGI1, TGM6, FUT3, TRIM10, LOC100128239, KRTAP19-8, PPIP5K1, B3GNT4, EPCAM, MPZL2, LAMA1, RTN4RL1, FAM151A, MYPN, CKB, KIAA1211, ACSL5, FUT6, SLC29A4, C4orf19, AHNAK2, TMEM8B, ECHDC2, TSTD1,	CYP27B1, CSF3R, CLEC4A, TNFRSF10C, ATHL1, FMO3, NFKBIZ, FAM26F, CD74, CCDC109B, CLDN14, LOC100133331, EFEMP1, SLC31A2, ISL2, MMP9, C22orf34, SLIT1, IL2RB, IFI44, TLR10, CXCL9, C10orf53, LMCD1, PHGDH, SIRPB1, NINJ1, NLRC3, ABTB1, LOC731275, STAP1, PARP14, LOC100128429, C2, BICD1, SEZ6, ALOX5AP, C21orf96, ICAM4, NCF1, GFPT2, LOC100127886, AGER, ODZ3, FBLN7, FADS3, CCDC3, CXorf65, SOCS3, CCL3L3, CLNK, LOC221442, SIK1, IL31RA, TNFSF14, ANKRD6, SH2D2A, ABCD2, HS3ST3A1, LOC348120, GOLGA6A, NCF2, FAM122C, TLR1, ANKRD26P1, LARP6, ITGB2, PLA2G2C, CALCRL, ETS1, LOC285965, AOX1, LRRC4, FAM176A, KLRG1, OR1J2, SNAI3, TNFRSF1B, HOXB2, GRAMD1B, SH3GL3, EGFLAM, DNHD1, ENPP2, FCGR2C, KCNA3, IL17RA, ENTHD1, CNTLN, REG1A, TOX2, TNFSF12, FAM184A, EFHA2, CD1C, GNN, PTPLAD2, ZNF267, MEOX2, HCG27, SAT1, RGL4, IL19, LOC84856, CCR6, TFAP2C, FUT8, LOC100128242, BTLA, FGF11, NEGR1, CNR2, ADAMTS18, SUSD3, COL7A1, LINC00173, MAGED4B, LILRA1, C4B, JSRP1, ESPNL, SNAI1, NLRP7, C2CD3, PSTPIP2, THBS2, COL1A2, TMEM158, LOC729737, CXCL13, REM2, LRRC10, DOK5, FADS2, LOC100129195, ARSG, KRTAP3-1, LOC729860, NLRC4, AKT1S1, S1PR1,

			TSPAN4, RAPGEF4, SBSN, TTN, MNDA, ACTL7A, SAMHD1, PRRX2, PRKCB, LILRB2, NOTCH3, SIGIRR, IRAK3, ZIK1, FLJ22447, ZFHX4, DDIT4, HSD11B1, ACSBG1, CD226, ADM2, LOC93444, C15orf53, C21orf7, C10orf114, DKFZp451A211, CNTRL, GIMAP2, ZNF331, CRTAM, GPR114, CLU, HLA-DOA, IL5RA, TMEM91, DYSF, ADCY4, RRAD, IL11, PDGFRB, TSHZ2, LOXL2, SLC7A7, TIMD4, SYTL3, IFIT2, CABYR, LOC100508384, LOC400968, RASGRF1, CCL3, APOBEC3H, LOC100289090, LZTS1, LST1, KAL1, HSF5, MGC39372, PHLDB2, KRTAP23-1, LOC100130298, SRMS, FAM27A, UBQLNL, LOC606724, GOLGA8F, CHIT1, FCGR1B, MMP14, MAPK12, LOC100240735, FCRLA, C4orf47, CCDC88B, SNPH, TDRD9, HVCN1, CREM, JAKMIP3, C9orf21, OR7A17, TWIST1, NRIP3, LOC100506310, WARS, SLC9A7P1, H19, TGFB2, IL18RAP, IFI44L, LOC151657, OBFC2A, JAKMIP1, GJC1, VEGFC, PRF1, REG1B, VCAM1, HK3, IFIT3, KIAA1462, MANEA, OR1E1, CD200, EGR3, RIPK2, IRF7, LOC100131831, IL10, FAM129C, C13orf33, EBF3, MTMR1, FCER1G, ADAMTS2, LRMP, SAA1, SLC16A6, C8orf31, GPR84,

Figure S6 Gene term enrichment analysis of the 2789 DEGs of dataset GSE111761 (RE vs NRE)

(A) Pathway analysis:

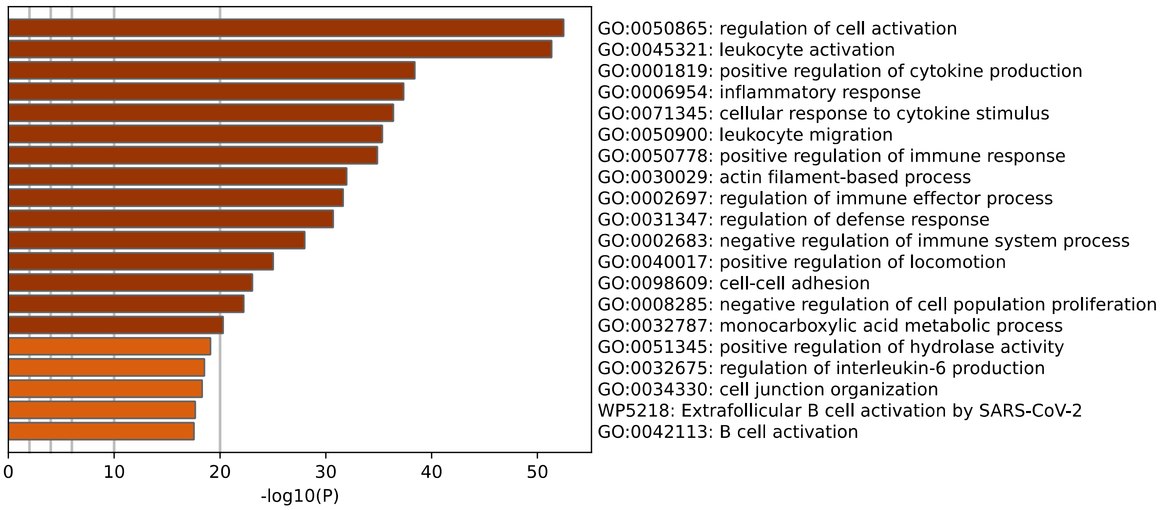

(B) Transcription factors:

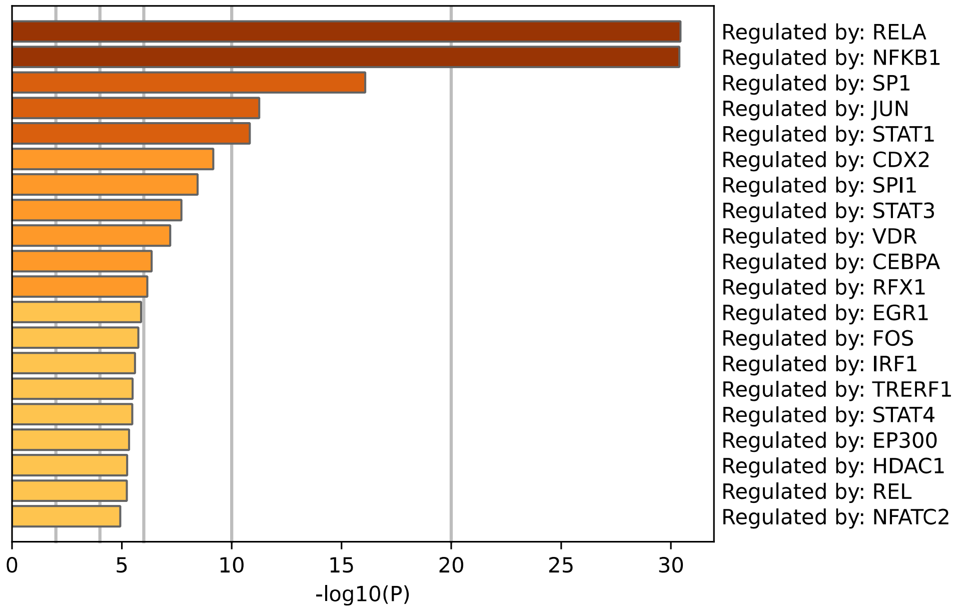

Supplement: Supplementary file 1 [file life-13-00680-s001.zip › life-2211618-supplementary.pdf]
